# Suppressing thermal quenching via defect passivation for efficient quasi-2D perovskite light-emitting diodes

**DOI:** 10.1038/s41377-022-00761-4

**Published:** 2022-03-23

**Authors:** Dezhong Zhang, Yunxing Fu, Hongmei Zhan, Chenyang Zhao, Xiang Gao, Chuanjiang Qin, Lixiang Wang

**Affiliations:** 1 State Key Laboratory of Polymer Physics and Chemistry, Changchun Institute of Applied Chemistry, Chinese Academy of Sciences, Changchun, 130022 China; 2 School of Applied Chemistry and Engineering, University of Science and Technology of China, Hefei, 230026 China

**Keywords:** Organic LEDs, Optoelectronic devices and components

## Abstract

Emission thermal quenching is commonly observed in quasi-2D perovskite emitters, which causes the severe drop in luminescence efficiency for the quasi-2D perovskite light-emitting diodes (PeLEDs) during practical operations. However, this issue is often neglected and rarely studied, and the root cause of the thermal quenching has not been completely revealed now. Here, we develop a passivation strategy via the 2,7-dibromo-9,9-bis (3′-diethoxylphosphorylpropyl)-fluorene to investigate and suppress the thermal quenching. The agent can effectively passivate coordination-unsaturated Pb^2+^ defects of both surface and bulk of the film without affecting the perovskite crystallization, which helps to more truly demonstrate the important role of defects in thermal quenching. And our results reveal the root cause that the quenching will be strengthened by the defect-promoted exciton-phonon coupling. Ultimately, the PeLEDs with defect passivation achieve an improved external quantum efficiency (EQE) over 22% and doubled operation lifetime at room temperature, and can maintain about 85% of the initial EQE at 85 °C, much higher than 17% of the control device. These findings provide an important basis for fabricating practical PeLEDs for lighting and displays.

## Abstract (web summary)

Highly efficient quasi-2D perovskite light-emitting diodes with suppressed thermal quenching by avoiding defect-promoted exciton-phonon coupling were prepared via a phosphate fluorene passivation agent.

## Graphical abstract

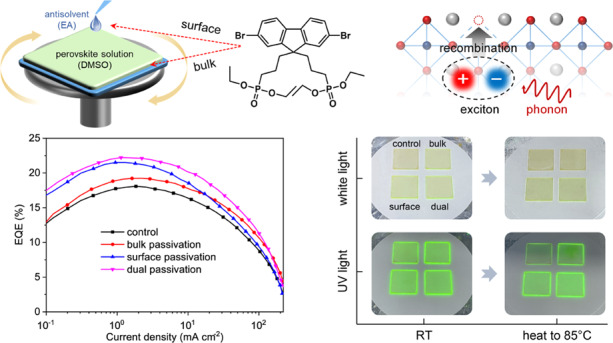

## Introduction

Lead halide perovskites have made rapid progress in photonic and optoelectronic applications in light of their easy preparation, defect tolerance, and excellent photoelectric properties^1–6^. The quasi-two-dimensional (quasi-2D) perovskite with reduced dimension can construct the multiple quantum wells to obtain confinement and dielectric shielding^7–10^, thus improving the exciton binding energy and enabling the photoluminescence quantum yields (PLQYs) to approach 100%^11–13^. The emission behavior of quasi-2D perovskites is determined by their unique recombination kinetics. The management of singlet and triplet excitons in quasi-2D perovskites has been discussed^10^, which is fundamental to the design of efficient perovskite light-emitting diodes (PeLEDs) and laser gain media. Benefiting from the well matching with typical optical microcavities, the optical feedback can be provided to the perovskite quasi-2D emitters^14,15^, and our group first achieved the stable room temperature (RT) continuous photo-induced perovskite laser^16^. Meanwhile, extensive works based on quasi-2D perovskites have been performed to improve the performance of PeLEDs, obtaining highly efficient green and red devices with the external quantum efficiencies (EQEs) exceeding 20%^17–21^, and blue PeLEDs more than 10%^22,23^. The efficient quasi-2D perovskite emitters can be prepared by simple one-step solution processing with tunable mechanical characteristics and demonstrate great potential for low-cost and flexible lighting and display^24,25^. However, the quasi-2D perovskites as the emitters usually suffer from a problem of thermal quenching that is easy to be ignored. Actually, heat generation is an unavoidable factor during the device operation. As reported, the thermal conductivity of organic-inorganic hybrid perovskites is very low^26–28^, which is similar to some typical organic semiconductors^29^. In practical applications, the junction temperature of the electroluminescent PeLED can reach 85 °C or even higher due to the Joule heat in the electric field^30–32^. So, it is necessary to improve the dissipation of heat from the active region of the device and inhibit the thermal quenching of the perovskite emitter itself.

The photoluminescence (PL) thermal quenching has been found in 3D, quasi-2D, and nanocrystals (NCs) perovskites^33–37^. Some aspects are considered to be the potential reasons for this: the thermally activated carrier trapping^33,38^, more excitation energy loss of the luminescence center in the form of lattice vibration due to the temperature increasing^39^, and the thermal degradation caused by the insufficient stability of perovskites^30,40,41^. Recently, considerable works have been conducted for understanding the thermal quenching behavior of perovskite NCs, and several results have demonstrated that the thermal quenching is related to the defects in perovskite NCs. Regulating perovskite components or incorporating additives can minimize the defects, accompanied by the suppression of thermal quenching^33–35^. However, the crystallization of perovskite NCs was inevitably affected during their optimization, thus the crucial mechanisms between defects and thermal quenching need to be further explored. As for the quasi-2D perovskites, the thermal quenching and its inhibition have not been systematically reported yet. Meanwhile, considering their unique luminescence mechanism with energy funnel, the effect of defects in emitting region and energy transfer region of the quasi-2D perovskite film on device performance and thermal stability should be explored.

Here, to reveal the direct correlation between thermal quenching and defects, we develop a phosphate fluorene passivation agent with functional groups that can effectively passivate the coordination-unsaturated Pb^2+^ defect, while the crystallization of quasi-2D perovskite is not affected at all after passivation. And the flexible solubility of the agent enables it to be selectively used to achieving surface passivation and bulk passivation for different energy landscapes in quasi-2D perovskite film. The passivation for the surface as the emitting region can improve the external quantum efficiency (EQE) and inhibit the thermal quenching more effectively, while the bulk passivation contributes greatly to the operation stability of PeLEDs at RT. Based on the dual passivation strategy, the PeLED achieves a maximum EQE of 22.2% and doubled operation lifetime. Meanwhile, the thermal quenching is significantly suppressed. The device with dual passivation can maintain about 85% of the initial EQE at 85 °C. And we reveal that the defects will induce the thermal quenching by strengthening the exciton-phonon interaction in quasi-2D perovskite.

## Results

**Fig. 1:**
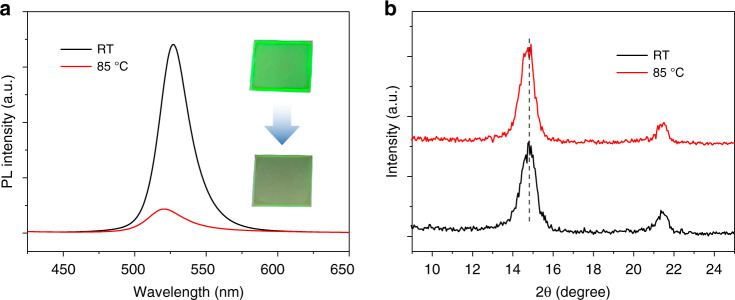
Emission thermal quenching. **a** PL spectra and photographs of a quasi-2D perovskite film excited at RT and 85 °C. **b** XRD patterns of perovskite film measured at RT and 85 °C

In this work, the Ruddlesden-Popper (RP) phase quasi-2D perovskite PEA_2_FA_n-1_Pb_n_Br_3n+1_ (PEA is phenylethylammonium, FA is formamidinium) with stoichiometric of *n* = 5 is employed as the emitters, and the structural diagram of the quasi-2D perovskite is shown in Fig. S1. The obvious thermal quenching of perovskite emitters can be commonly noticed. In Fig. 1a, the PL intensity of quasi-2D perovskite film is significantly decreased at 85 °C, which can be intuitively observed from the emission images. The weakened PL intensity of the emitter will make the device no longer work efficiently at 85 °C, which is a serious and urgent problem to be solved. Meanwhile, the blue shift of PL peak can be observed, which is related to the lattice expansion and has been verified by the slight shift of heated X-ray diffraction (XRD) peaks in Fig. 1b. Actually, the calculated lattice constants are 5.90 Å and 5.92 Å at RT and 85 °C, respectively, thus the lattice expansion is relatively weak. So, the thermal quenching is not caused by the lattice distortion or phase transition^36,42^. Some works infered that the thermal quenching of perovskite is related to defects^33,38,40^. And we intend to study the direct relationship between defects and thermal quenching by developing a passivation strategy.

**Fig. 2:**
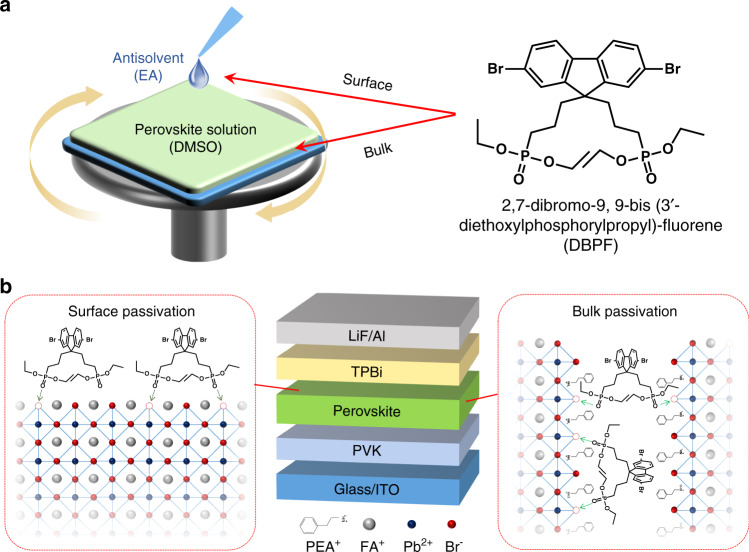
Methods and mechanisms of defect passivation in quasi-2D perovskite film. **a** Preparation of quasi-2D perovskite film and molecular chemical structure of DBPF passivation agent. **b** Schematics of device structure and passivation mechanisms

We designed and developed a passivation agent for quasi-2D perovskite emitter, which can passivate the defects without affecting the crystallization. Figure 2a presents the preparation method of quasi-2D perovskite film, as well as the chemical structure of passivation agent of 2,7-dibromo-9,9-bis (3′-diethoxylphosphorylpropyl)-fluorene (DBPF). The synthetic route of DBPF is shown in Fig. S2, and the detail synthesis methods are provided in materials and methods part. For the passivation agent of DBPF, alkyl phosphates with lower coordination strength were selected as passivation functional groups, which can avoid strong complexation with Pb ions in the precursor solution but will effectively passivate the coordination-unsaturated Pb^2+^ defect in the perovskite films. The DBPF can cleverly realize efficient defects passivation, while without affecting the perovskite crystallization behavior, which is helpful to explore the direct relationship between various properties and defects. Based on nonpolar fluorene and polar phosphate groups, the DBPF can be dissolved into perovskite precursor solution (dimethyl sulfoxide, DMSO) and antisolvent (ethyl acetate, EA) to achieve bulk passivation and surface passivation, which is benefit to further explore the relationship between defects in different landscapes and device performance including thermal stability. The schematics of device structure and passivation mechanism are plotted in Fig. 2b. The bulk and surface passivation for the perovskite film are based on the same mechanism of coordinating with Pb^2+^, but their impacts on device performance and thermal quenching may not be consistent. Thus, different passivation strategies were performed to further fabricate PeLEDs, including bulk passivation, surface passivation, and dual passivation, and the detail preparations are provided in the materials and methods part.

**Fig. 3:**
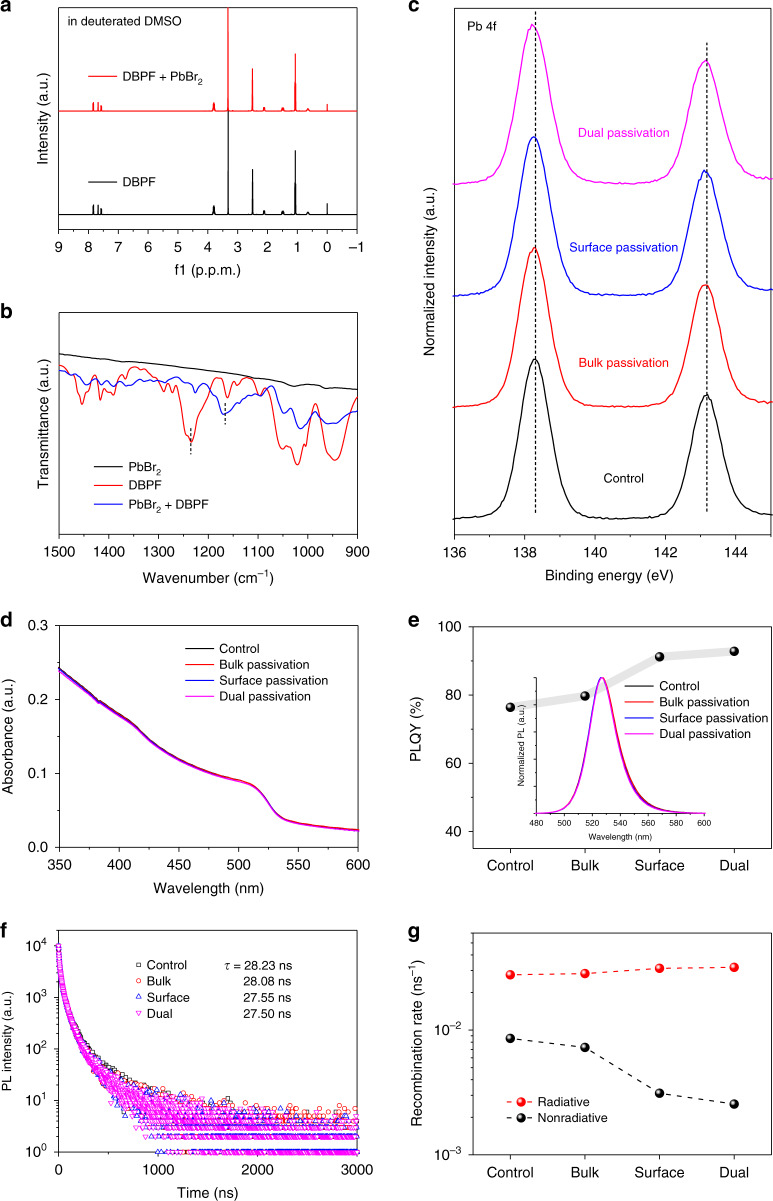
Working mechanism of DBPF. **a** ^1^H NMR spectra of DBPF without and with PbBr_2_ dissolved in deuterated DMSO. **b** FTIR spectra of PbBr_2_, DBPF, and DBPF with PbBr_2_. **c** XPS of Pb 4 f peaks, **d** light absorbance spectra, **e** PLQY values and normalized PL spectra, **f** PL-decay curves, and **g** recombination rates of perovskite films without and with different passivation strategies

To verify the interaction between DBPF and non-coordinating Pb^2+^, the nuclear magnetic resonance (NMR) spectra were first measured to analyze the effect of DBPF in the precursor solution. The deuterated DMSO is used as the solvent to test the NMR spectra of DBPF without and with PbBr_2_, as shown in Fig. 3a and Fig. S3 of the ^1^H and ^31^P NMR spectra, and no obvious change of the chemical shifts was observed. This may be due to the weaker coordination between Pb^2+^ and DBPF than DMSO. While the interaction between DBPF and PbBr_2_ is detected by the Fourier transformed infrared spectroscopy (FTIR) measurements of solid powder (Fig. 3b). The stretching of P = O bonds of DBPF at ca.1230 cm^−1^ exhibits a clear shift with the presence of PbBr_2_, which fully demonstrates that the DBPF can coordinate with Pb^2+^ and will passivate the defects for perovskite layer^7,43–45^. Above results also indicate that the DBPF can’t demonstrate the coordination ability with Pb^2+^ in DMSO solvent, thus the crystal formation of quasi-2D perovskite layer will not be changed with the incorporation of DBPF. It is consistent with the XRD results in Fig. S4. And all films have approximate morphology and surface roughness as shown in Fig. S5. The X-ray photoelectron spectroscopy (XPS) measurements of different perovskite films are carried out, as exhibited in Fig. 3c. After passivation, the weak shifts of Pb 4 f peaks to low binding energy are all observed. The shift is attributed to the change in the electron cloud density, indicating the interaction between the passivation agent and perovskite^18,46^, simultaneously proving that the bulk and surface of the perovskite layer can be electrostatically passivated by DBPF. In Fig. 3d, the perovskite layers exhibit almost the same light absorbance due to their similar crystallization behavior and the wide bandgap (4.7 eV, HOMO = − 5.99 eV, LUMO = − 1.29 eV) of DBPF.

Figure 3e lists the PLQY values of quasi-2D perovskite films with different passivation strategies, and inset is the normalized PL spectra. It is noteworthy that the PLQY of perovskite films after the surface and dual passivation are obviously enhanced, while the improvement of the perovskite layer with bulk passivation is relatively weak. This is because that the main emitting region of the quasi-2D perovskite film is near the surface that is rich in higher-order landscapes, which has been conformed in our previous work^47^. Though the defects in bulk have potential negative effects to induce quenching, it has been revealed that the energy transfer is faster than defect trapping^48,49^, thus bulk passivation demonstrates little contribution to PLQY enhancement. At the same time, there is no obvious shift of PL peaks, and the shoulder of PL peaks at ca. 550 nm decreases a little after passivation, which can be attributed to the weakened defect-induced recombination. The corresponding PL-decay curves were measured and shown in Fig. 3f, and the average lifetime (*τ*) is obtained by three index fitting. After passivation, the *τ* is slightly shortened, which represents the change in recombination behaviors. Based on the above results, we calculate the radiative and nonradiative recombination rate (*k*_r_, *k*_nr_) of the perovskite films using the equation of PLQY = *k*_r_/(*k*_r_ + *k*_nr_) and *τ* = (*k*_r_ + *k*_nr_)^−1^ in Fig. 3g^50,51^. The *k*_nr_ is obviously decreased after surface and dual passivation, and the corresponding *k*_r_ is slightly improved. These evidences can fully verify that the nonradiative recombination is closely related to the defects in quasi-2D perovskites, and defect passivation for the emitting region of the film surface is the key to weaken the nonradiative recombination and improve the PLQY.

**Fig. 4:**
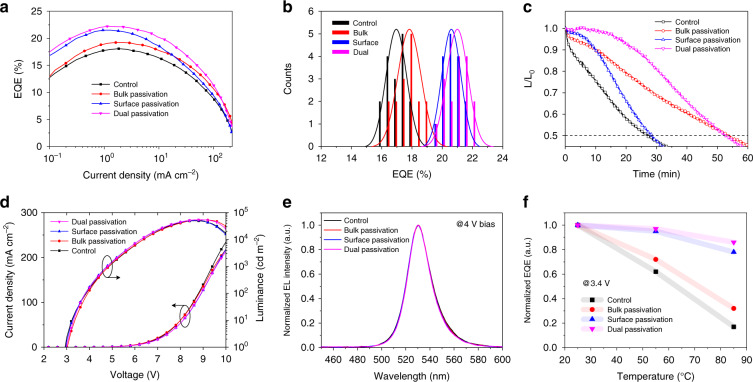
Optoelectronic characteristics of the quasi-2D PeLEDs. **a** Current density-EQE curves, **b** histograms of peak EQEs, **c** operation stability at a initial luminance of 100 cd m^−2^, **d** current density-voltage-luminance characteristics and **e** EL spectra of PeLEDs based on perovskite emitters without and with different passivation strategies. **f** Normalized EQE comparison of PeLEDs from RT to 85 °C

We investigated the role of defect passivation in performances enhancement for PeLEDs at RT first. The device structure and corresponding cross-sectional SEM image are plot in Fig. S6, as well as the energy levels distribution. As shown in Fig. 4a of the current density-EQE curves, the peak values after surface passivation and dual passivation reach 21.5% and 22.2% respectively, higher than 18.1% of the control device and 19.3% of bulk passivation device. The bulk and surface passivation processes were optimized, respectively, as shown in Figs. S7 and S8 in the supplementary information. And the EQE cartograms for devices with different passivation strategies are presented in Fig. 4b. It is worth noting that the surface passivation contributes greatly to the improved EQE peak values, which is attributed to the enhanced PLQY of emitters. And the EQE peak can be obtained at lower current density, which is also observed in some previous reports using the surface passivation^18,44^. This could be due to that the nonradiative recombination induced by defects is more competitive under low injection conditions, which can be perfectly suppressed by surface passivation. While under higher current density injection conditions, the relaxation of recombination originating from defects, will weaken the competitiveness of the defect-induced nonradiative recombination^52–54^.

Though the bulk passivation can also remarkably decrease the trap density in the perovskite film obtained from the current density-voltage characteristics of hole-only devices in Fig. S9, the defects in bulk have no obvious effect on peak EQE due to the fast energy transfer in the quasi-2D film. In additon, the similar efficiency roll-off can be observed before and after surface passivation. And when the bulk passivation strategy is introduced, the roll-off will be slightly suppressed. We think that the bulk passivation can suppress the ion migration by filling vacancies at grain boundary, thus slowing down the degradation of device performance. In Fig. S10, the smaller hysteresis also verifies that the ion migration is suppressed after bulk passivation, which is potential for long time working of devices. As expected in Fig. 4c, the operation stability for the quasi-2D PeLEDs at the initial luminance of 100 cd m^−2^ is measured, and the operation lifetime of PeLEDs is doubled by bulk passivation and dual passivation. The two devices obtain slightly higher brightness under a larger bias, as shown in Fig. 4d. To sum up, the surface passivation can significantly improve the EQE peak values of PeLEDs, and the bulk passivation will make outstanding contribution to the operation stability. The performances of all devices at RT are summarized in Table S1.

The electroluminescence (EL) peak values of devices before and after passivation are the same, as shown in Fig. 4e. Meanwhile, we investigated the spectral stability of the optimal device with dual passivation, and Fig. S11 shows the normalized EL spectra from 4 V to 10 V. The device demonstrates good spectral stability, and only a slight blue shift is observed when exceeding a high bias of 9 V, which is related to the landscapes distribution and recombination position in quasi-2D perovskite film^42^.

We have further compared the EQE of devices at RT and higher temperature, and the normalized EQE comparison of PeLEDs from RT to 85 °C is plotted in Fig. 4f. The device with dual passivation can maintain 85% of the initial EQE, which is much higher than 17% of the control device. The surface passivation can more effectively suppress the thermal quenching than bulk passivation. Our passivation strategy improves the performance of the device working at high temperature, and it is a direct evidence to prove that the thermal quenching is closely related to the defects.

**Fig. 5:**
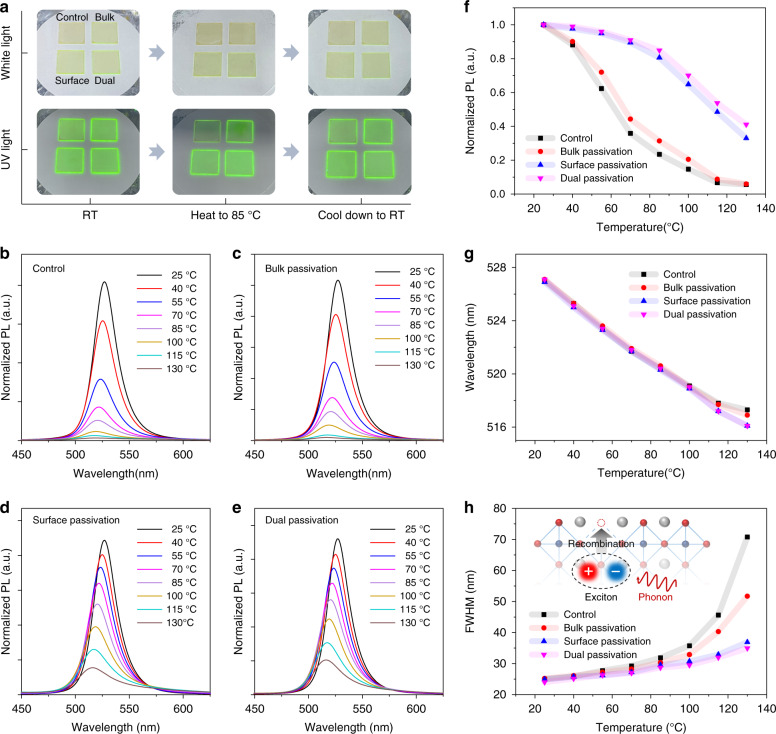
Impacts of passivation on thermal quenching characteristics of quasi-2D perovskite. **a** Thermal quenching and recovery processes of quasi-2D perovskite films under white and UV light. Temperature-dependent PL spectra of perovskite films **b** without, **c** with bulk, **d** with surface, and **e** with dual passivation. **f** Integrated PL intensities, **g** wavelength and **h** FWHM of PL peaks for perovskite films without and with dual passivation. Inset of (**h**) is the diagram of exciton-phonon interaction and nonradiative recombination promoted by defects

To deeply investigate the root cause of the defect-induced thermal quenching, the thermal quenching behavior of perovskite films is monitored. In Fig. 5a, when the perovskite films are heated to 85 °C, the PL is obviously quenched. While after passivation, the thermal quenching is apparently suppressed, followed by the emission recovery reversibly when cooling down to RT. Corresponding PL spectra of all samples at different conditions are shown in Fig. S12. Meanwhile, the surface passivation is superior to bulk passivation to inhibit thermal quenching, which confirms that the main energy loss occurs in the exciton recombination process rather than the energy transfer process. Based on dual passivation, the film demonstrates the optimal thermal stability. Figures 5b–e show the temperature-dependent PL spectra of films, and corresponding integrated PL intensities are summarized in Fig. 5f. After the dual passivation, the PL remains above 80% at 85 °C, which is consistent with the EQE of device. And at 115 °C, the optimal film still preserves 50% of the initial PL intensity, while the control film has been almost completely quenched. Suppressed thermal quenching from defect passivation can indicate that the heat in form of the phonon accelerates the defect-induced nonradiative recombination.

To understand the participation pathway of heat, we clearly analyze the position and full width at half maximum (FWHM) of peaks of temperature-dependent PL spectra, as shown in Fig. 5g, h. The PL peak wavelength varies approximate linearly, and the similar shifts of EL peaks of devices are also obtained in Fig. S13. The lattice expansion of films without and with passivation are approximate, as plotted in Fig. S14. To explore whether the heating process produces phase transition of quasi-2D perovskite films, the temperature-dependent PL peak energies of all films are plotted in Fig. S15. The PL peak energies for each film are almost the same in both heating and cooling processes, and no hysteresis is observed in the transition temperature between the heating and cooling cycle. So, no phase transition occurs within the test temperature range^55,56^, which further eliminates the correlation between thermal quenching and crystallization characteristics. In Fig. 5h, the FWHM of PL spectra are broadened as temperature increases for all samples, which is attributed to the recombination energy fluctuation caused by the exciton-phonon coupling^55,57,58^. And it is noteworthy that more significantly broadened FWHM for the control and bulk passivation films reflects the stronger exciton-phonon coupling strengthened by more defects at the film surface. According to above results, it can be concluded that defect-induced exciton-phonon coupling in the emitting region of the quasi-2D film, will aggravate the nonradiative recombination of the exciton, leading to severe thermal quenching. When the temperature is higher than 115 °C, the wavelength of PL peak deviates from linearity and the FWHM increases sharply for control and bulk passivation films, indicating that there should be another quenching mechanism involving higher-energy phonons. As the perovskite layers were annealed at 130 °C and returned to RT, the PL spectra in Fig. S16 show that the emission intensities of control and bulk passivation films cannot return to their initial values, while the perovskites with surface and dual passivation can maintain more than 98% of the initial PL intensity. The photographs in Fig. S17 also show the samples cooling down to RT after a period of 130 °C annealing, and the perovskites can be protected by surface passivation and dual passivation with maintained excellent luminescence properties. Therefore, the higher-energy phonons will lead to the irreversible damage of the emitting region in quasi-2D perovskite films, and this degradation can also be inhibited by defect passivation. In addition, it is worth mentioning that the thermal quenching can be suppressed by the defect passivation, but the operation stability of devices at 85 °C is still poor, as shown in Fig. S18. It seems to indicate that the severe ion migration at high temperature is difficult to be suppressed by the strategy of coordination passivation. We find that passivating the defects and anchoring the free-ions are more effective methods by incorporating additives with phosphonic acid groups, and molecular design and corresponding working mechanisms are currently exploring in depth, which will be further demonstrated in our subsequent studies.

## Discussion

In this work, we have revealed that defects can cause stronger exciton-phonon coupling, which induces the thermal quenching in quasi-2D perovskite. And the thermal quenching can be significantly suppressed by employing the DBPF due to the interaction with coordination-unsaturated Pb^2+^ defects. Meanwhile, research results demonstrate the correlation between passivation for different landscapes and device performances, as well as the thermal quenching. As a result, over 22% of EQE and doubled operation lifetime are obtained for PeLEDs based on dual passivation at RT, and the PeLEDs still maintain 85% of the initial EQE at high temperature of 85 °C. The findings in this work pave a novel way for realizing efficient and stable PeLEDs, and hold promise for the realization of high-efficiency PeLEDs at high temperatures.

## Materials and methods

### Materials

PVK was purchased from Sigma Aldrich. FABr, PEABr, PbBr_2_, and MACl were purchased from Xi’an Polymer Light Technology Corp. TPBi and LiF were purchased from Jilin Oled Material Technology Corp. DMSO and ethyl acetate were purchased from J&K Scientific Corp. All the chemical materials were directly used without any further purifications.

### Synthesis of DBPF

A mixture of 2,7-dibromofluorene (6.5 g, 20 mmol), 1,3-dibromopropane (30 mL), tetrabutylammonium bromide (0.1 g), and sodium hydroxide (50% w/w) aqueous solution (30 mL) was stirred at 70 °C, and refluxing for 10 h. After cooling down to RT, the mixture was added to dichloromethane for extraction. The organic layer was washed with deionized water and brine. Then the separated organic layer was dried over anhydrous Na_2_SO_4_ and filtered. The dichloromethane was removed under vacuum, and the superfluous 1,3-dibromopropane was distilled under vacuum. The residue was purified by flash column chromatography with petroleum ether to get 2,7-dibromo-9,9-bis(3′-bromopropyl)-fluorene (5.8 g, 47%) as a white powdered solid.

A solution of 2,7-dibromo-9,9-bis(3′-bromopropyl)-fluorene (2.83 g, 5 mmol) in triethyl phosphite was heated to 140 °C for 18 h in nitrogen atmosphere. Excess triethyl phosphate was distilled under vacuum. The residue was purified by eutral Al_2_O_3_ using ethyl acetate/petroleum ether (1:1) as eluent to obtain white granular microcrystals (2.8 g, 82%).

### Preparation of quasi-2D perovskite layers

All quasi-2D perovskite layers are obtained by spin-coating. The precursor solution is prepared by mixing the PbBr_2_, FABr, PEABr, and MACl with a molar ratio of 5:4:2:0.5 in DMSO, and the MACl acts as additive to improve the crystallization. The DBPF can be directly doped into perovskite precursor solution by mixing with other precursor components for bulk passivation. The 0.2 M (Pb^2+^ concentration) precursor solution is spin-coated at 8000 rpm for 30 s, and then 100 µL of ethyl acetate is poured onto the film during the spin-casting, followed by annealing on a hot plate at 85 °C for 20 min. As for the surface passivation, the DBPF is doped into ethyl acetate as the antisolvent. Finally, the quasi-2D perovskite layer is fabricated and the thickness is about 50 nm.

### Device fabrication

The indium tin oxide (ITO)-coated glass substrates are sequentially cleaned in detergent, distilled water, acetone and isopropanol by an ultrasonic cleaner. The pre-cleaned substrates are ultraviolet ozone treated for 30 min to make the surface hydrophilic, and then transferred into a nitrogen-filled glove box. PVK solution (10 mg ml^−1^ in chlorobenzene) is spin-coated at 4000 rpm for 40 s and the films are baked at 120 °C for 30 min. After that, the perovskite film is deposited on PVK layer. Ultimately, TPBi (50 nm), LiF (1 nm), and Al (100 nm) are deposited by thermal evaporation, respectively. The active device area is 0.08 cm^2^.

### Material and device characterizations

UV-Vis absorption spectra of quasi-2D perovskite layers were obtained by a Perkin-Elmer Lambda 35 UV-vis spectrometer. PL spectra and PLQY at RT were measured by a HORIBA FL3C-111 spectrofluorometer equipped with an integrating sphere. XRD curves were obtained using a Rigaku SmartLab diffractometer. XPS measurements were performed by an ESCALAB 250 spectrometer. FTIR was measured on a Thermo Scientific Nicolet iS50. SEM images were obtained from a JEOL JSM‐7500 field‐emission SEM. Topography images of the corresponding films were collected by SPI3800N AFM. The NMR spectra were recorded by a Bruker Avance NMR spectrometer (500 MHz). The transient PL-decay curves were measured with a DeltaFlex Modular Fluorescence Lifetime System (Horiba Scientific). PL thermal quenching was recorded by a fiber-optic spectrometer in the glove box. All the PeLEDs performance tests were performed with an Ocean Optics LED integrating sphere test system in the glove box, and the schematic diagram of the test system is shown in Fig. S19.

## Supplementary information

Supplementary Information for Suppressing thermal quenching via defect passivation for efficient quasi-2D perovskite light-emitting diodes

## References

[1] Stranks SD (2013). Electron-hole diffusion lengths exceeding 1 micrometer in an organometal trihalide perovskite absorber. Science.

[2] Kovalenko MV, Protesescu L, Bodnarchuk MI (2017). Properties and potential optoelectronic applications of lead halide perovskite nanocrystals. Science.

[3] Han TH (2019). Interface and defect engineering for metal halide perovskite optoelectronic devices. Adv. Mater..

[4] Stranks SD, Snaith HJ (2015). Metal-halide perovskites for photovoltaic and light-emitting devices. Nat. Nanotechnol..

[5] Jena AK, Kulkarni A, Miyasaka T (2019). Halide perovskite photovoltaics: background, status, and future prospects. Chem. Rev..

[6] Sutherland BR, Sargent EH (2016). Perovskite photonic sources. Nat. Photonics.

[7] Yang XL (2018). Efficient green light-emitting diodes based on quasi-two-dimensional composition and phase engineered perovskite with surface passivation. Nat. Commun..

[8] Milot RL (2016). Charge-carrier dynamics in 2D hybrid metal-halide perovskites. Nano Lett..

[9] Wang NN (2016). Perovskite light-emitting diodes based on solution-processed self-organized multiple quantum wells. Nat. Photonics.

[10] Qin CJ (2020). Triplet management for efficient perovskite light-emitting diodes. Nat. Photonics.

[11] Sun CJ (2021). High-performance large-area quasi-2D perovskite light-emitting diodes. Nat. Commun..

[12] Cheng T (2020). Stoichiometry control for the tuning of grain passivation and domain distribution in green quasi-2D metal halide perovskite films and light-emitting diodes. Adv. Funct. Mater..

[13] Zhang L (2021). High-performance quasi-2D perovskite light-emitting diodes: from materials to devices. Light.: Sci. Appl..

[14] Vahala KJ (2003). Optical microcavities. Nature.

[15] Tsakmakidis KL (2016). Large spontaneous-emission enhancements in metallic nanostructures: towards LEDs faster than lasers. Opt. Express.

[16] Qin CJ (2020). Stable room-temperature continuous-wave lasing in quasi-2D perovskite films. Nature.

[17] Kong LM (2021). Smoothing the energy transfer pathway in quasi-2D perovskite films using methanesulfonate leads to highly efficient light-emitting devices. Nat. Commun..

[18] Chu ZM (2021). Perovskite light-emitting diodes with external quantum efficiency exceeding 22% via small-molecule passivation. Adv. Mater..

[19] Jiang YZ (2021). Reducing the impact of Auger recombination in quasi-2D perovskite light-emitting diodes. Nat. Commun..

[20] Ye YC (2021). Minimizing optical energy losses for long-lifetime perovskite light-emitting diodes. Adv. Funct. Mater..

[21] Fang ZB (2020). Dual passivation of perovskite defects for light-emitting diodes with external quantum efficiency exceeding 20%. Adv. Funct. Mater..

[22] Ren ZW (2021). High-performance blue perovskite light-emitting diodes enabled by efficient energy transfer between coupled quasi-2D perovskite layers. Adv. Mater..

[23] Chu ZM (2020). Large cation ethylammonium incorporated perovskite for efficient and spectra stable blue light-emitting diodes. Nat. Commun..

[24] Rolston N (2018). Effect of cation composition on the mechanical stability of perovskite solar cells. Adv. Energy Mater..

[25] Rolston N (2016). Mechanical integrity of solution-processed perovskite solar cells. Extrem. Mech. Lett..

[26] Pisoni A (2014). Ultra-low thermal conductivity in organic-inorganic hybrid perovskite CH_3_NH_3_PbI_3_. J. Phys. Chem. Lett..

[27] Qian X, Gu XK, Yang RG (2017). Thermal conductivity modeling of hybrid organic-inorganic crystals and superlattices. Nano Energy.

[28] Chen ZZ (2019). Merits and challenges of Ruddlesden-Popper soft halide perovskites in electro-optics and optoelectronics. Adv. Mater..

[29] Kim N (2005). Thermal transport properties of thin films of small molecule organic semiconductors. Appl. Phys. Lett..

[30] Li GH (2020). Stability of perovskite light sources: status and challenges. Adv. Optical Mater..

[31] Zou C (2020). Suppressing efficiency roll-off at high current densities for ultra-bright green perovskite light-emitting diodes. ACS Nano.

[32] Zhao LF (2020). Thermal management enables bright and stable perovskite light-emitting diodes. Adv. Mater..

[33] Liu MM (2021). Suppression of temperature quenching in perovskite nanocrystals for efficient and thermally stable light-emitting diodes. Nat. Photonics.

[34] Zhang Q (2020). Bifunctional passivation strategy to achieve stable CsPbBr_3_ nanocrystals with drastically reduced thermal-quenching. J. Phys. Chem. Lett..

[35] Zhang CX (2020). Core/shell perovskite nanocrystals: synthesis of highly efficient and environmentally stable FAPbBr_3_/CsPbBr_3_ for LED applications. Adv. Funct. Mater..

[36] He YR (2021). Perovskite light-emitting diodes with near unit internal quantum efficiency at low temperatures. Adv. Mater..

[37] Li XM (2016). CsPbX_3_ quantum dots for lighting and displays: room-temperature synthesis, photoluminescence superiorities, underlying origins and white light-emitting diodes. Adv. Funct. Mater..

[38] Diroll BT (2017). High-temperature photoluminescence of CsPbX_3_ (X = Cl, Br, I) nanocrystals. Adv. Funct. Mater..

[39] Liu SY (2020). Wide range zero-thermal-quenching ultralong phosphorescence from zero-dimensional metal halide hybrids. Nat. Commun..

[40] Wei Y, Cheng ZY, Lin J (2019). An overview on enhancing the stability of lead halide perovskite quantum dots and their applications in phosphor-converted LEDs. Chem. Soc. Rev..

[41] Quan LN (2020). Edge stabilization in reduced-dimensional perovskites. Nat. Commun..

[42] Lü XJ (2021). Regulating off-centering distortion maximizes photoluminescence in halide perovskites. Natl Sci. Rev..

[43] Li HS (2020). Intermolecular π-π conjugation self-assembly to stabilize surface passivation of highly efficient perovskite solar cells. Adv. Mater..

[44] Xu LM (2020). A bilateral interfacial passivation strategy promoting efficiency and stability of perovskite quantum dot light-emitting diodes. Nat. Commun..

[45] Zhao YP (2020). Molecular interaction regulates the performance and longevity of defect passivation for metal halide perovskite solar cells. J. Am. Chem. Soc..

[46] Xu WD (2019). Rational molecular passivation for high-performance perovskite light-emitting diodes. Nat. Photonics.

[47] Zhang DZ (2021). Domain controlling by compound additive toward highly efficient quasi-2D perovskite light-emitting diodes. Adv. Funct. Mater..

[48] Quan LN (2017). Tailoring the energy landscape in quasi-2D halide perovskites enables efficient green-light emission. Nano Lett..

[49] Yuan MJ (2016). Perovskite energy funnels for efficient light-emitting diodes. Nat. Nanotechnol..

[50] Xiao ZG (2019). Engineering perovskite nanocrystal surface termination for light-emitting diodes with external quantum efficiency exceeding 15%. Adv. Funct. Mater..

[51] Ribierre JC (2008). Triplet exciton diffusion and phosphorescence quenching in iridium(III)-centered dendrimers. Phys. Rev. Lett..

[52] Gil-Escrig L (2015). Efficient photovoltaic and electroluminescent perovskite devices. Chem. Commun..

[53] Kim YH (2021). Comprehensive defect suppression in perovskite nanocrystals for high-efficiency light-emitting diodes. Nat. Photonics.

[54] Byun J (2016). Efficient visible quasi-2D perovskite light-emitting diodes. Adv. Mater..

[55] Ni LM (2017). Real-time observation of exciton-phonon coupling dynamics in self-assembled hybrid perovskite quantum wells. ACS Nano.

[56] Sheikh T (2018). Possible dual bandgap in (C_4_H_9_NH_3_)_2_PbI_4_ 2D layered perovskite: single-crystal and exfoliated few-layer. ACS Energy Lett..

[57] Gong XW (2018). Electron-phonon interaction in efficient perovskite blue emitters. Nat. Mater..

[58] Peng SM (2020). Suppressing strong exciton-phonon coupling in blue perovskite nanoplatelet solids by binary systems. Angew. Chem. Int. Ed..

