# Supplementary material for: Suppressing thermal quenching via defect passivation for efficient quasi-2D perovskite light-emitting diodes
Source: Light Sci Appl. 2022 Mar 23;11:69. doi: 10.1038/s41377-022-00761-4 (PMC8943027; doi:10.1038/s41377-022-00761-4)
Supplement: Supplementary file 1 — Supplementary Information for Suppressing thermal quenching via defect passivation for efficient quasi-2D perovskite light-emitting diodes [file 41377_2022_761_MOESM1_ESM.docx]

**Supplementary Information**

**Suppressing thermal quenching via defect passivation for efficient quasi-2D perovskite light-emitting diodes**

Dezhong Zhang^1^, Yunxing Fu^1,2^, Hongmei Zhan^1^, Chenyang Zhao^1^, Xiang Gao^1^, Chuanjiang Qin^1,2✉^ and Lixiang Wang^1^

Correspondence: Chuanjiang Qin (cjqin@ciac.ac.cn)

^1^State Key Laboratory of Polymer Physics and Chemistry, Changchun Institute of Applied Chemistry, Chinese Academy of Sciences, Changchun 130022, China

^2^School of Applied Chemistry and Engineering, University of Science and Technology of China, Hefei 230026, China

These authors contributed equally: Dezhong Zhang, Yunxing Fu.


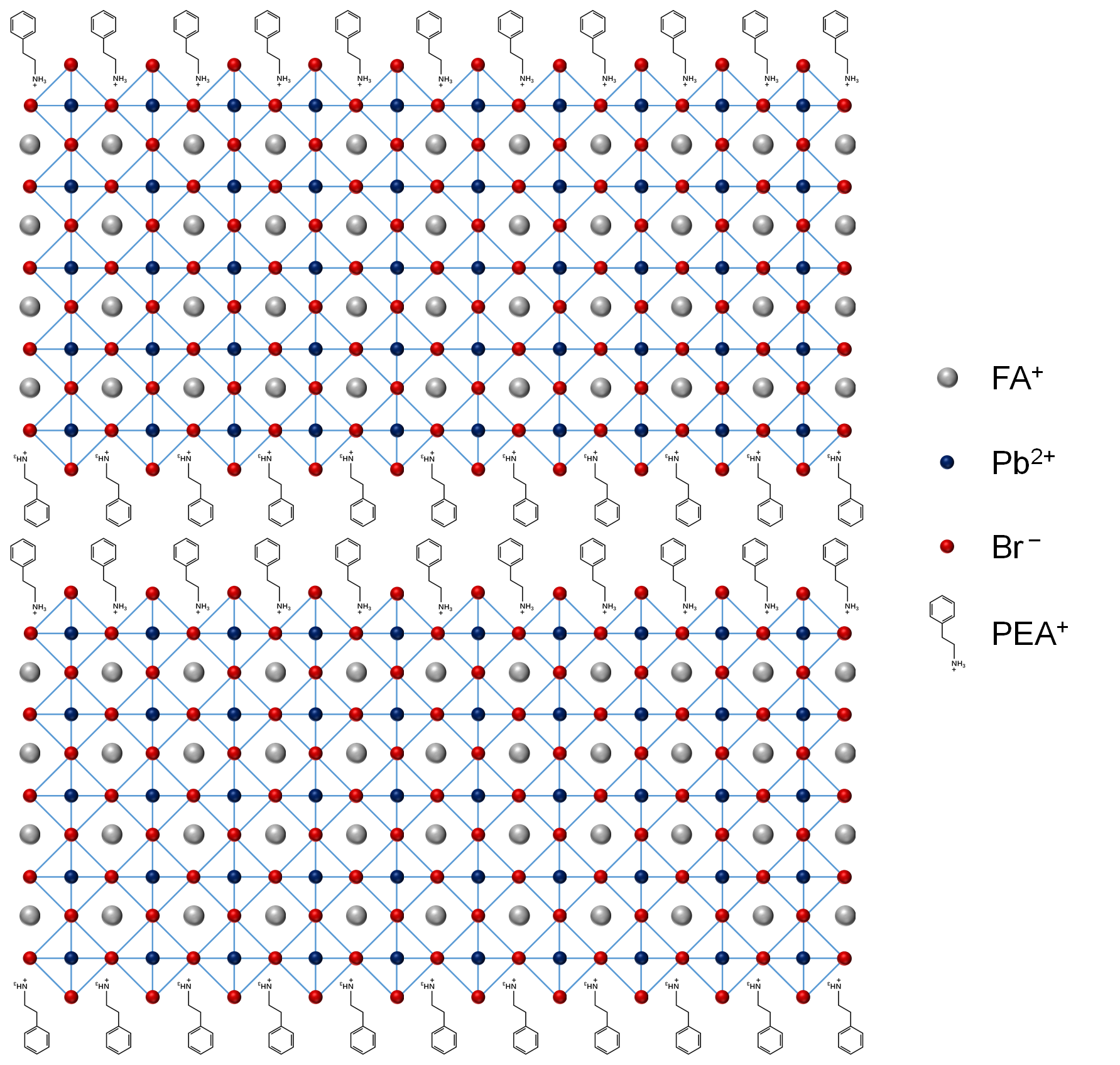


**Fig. S1.** Structural diagram of PEA_2_FA_n-1_Pb_n_Br_3n+1_ (n = 5) of quasi-2D perovskite employed in this work.

**Fig. S2** Synthetic Scheme for the preparation of DBPF.


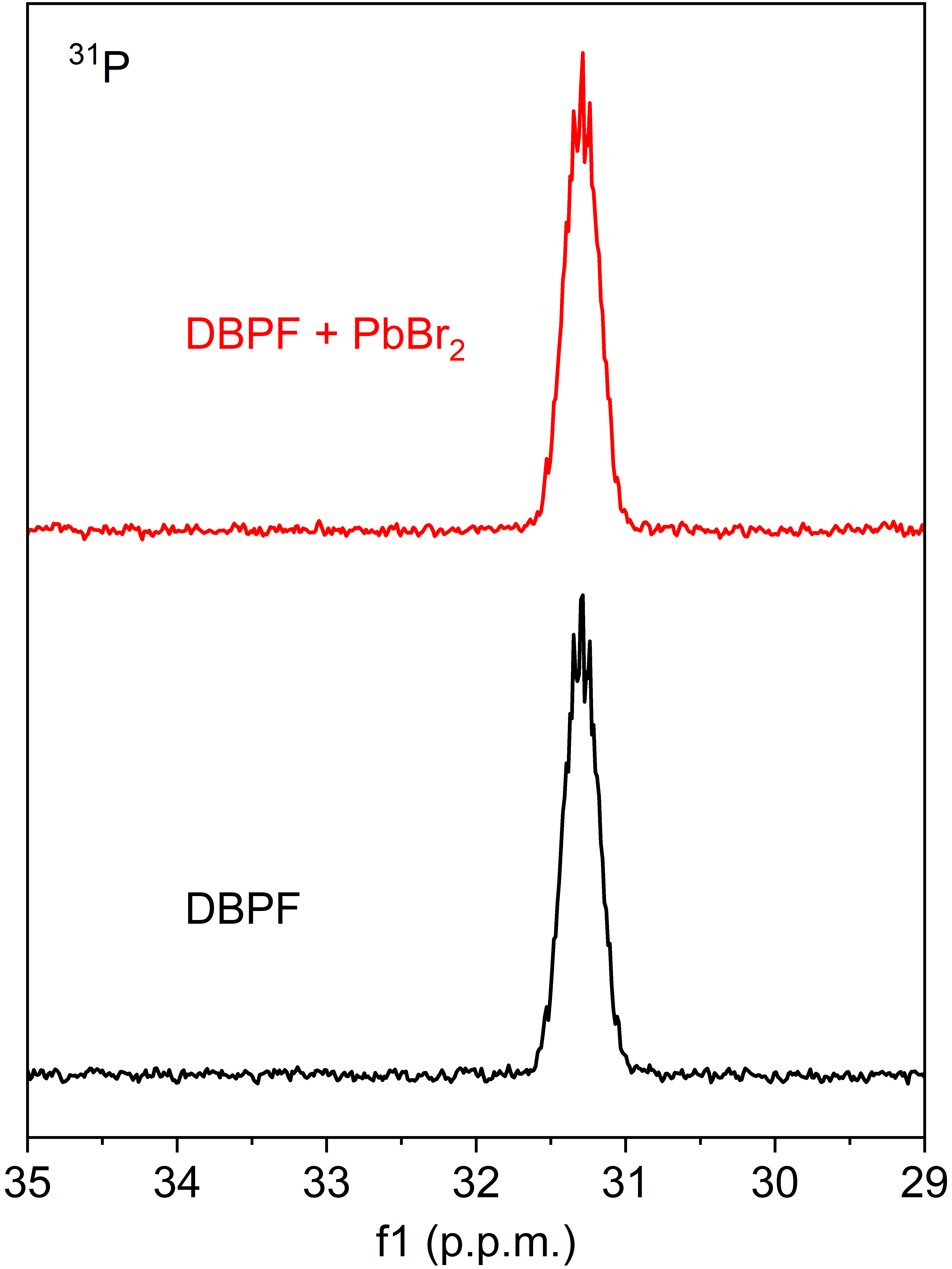


**Fig. S3** ^31^P NMR spectra of DBPF without and with PbBr_2_ dissolved in deuterated DMSO.


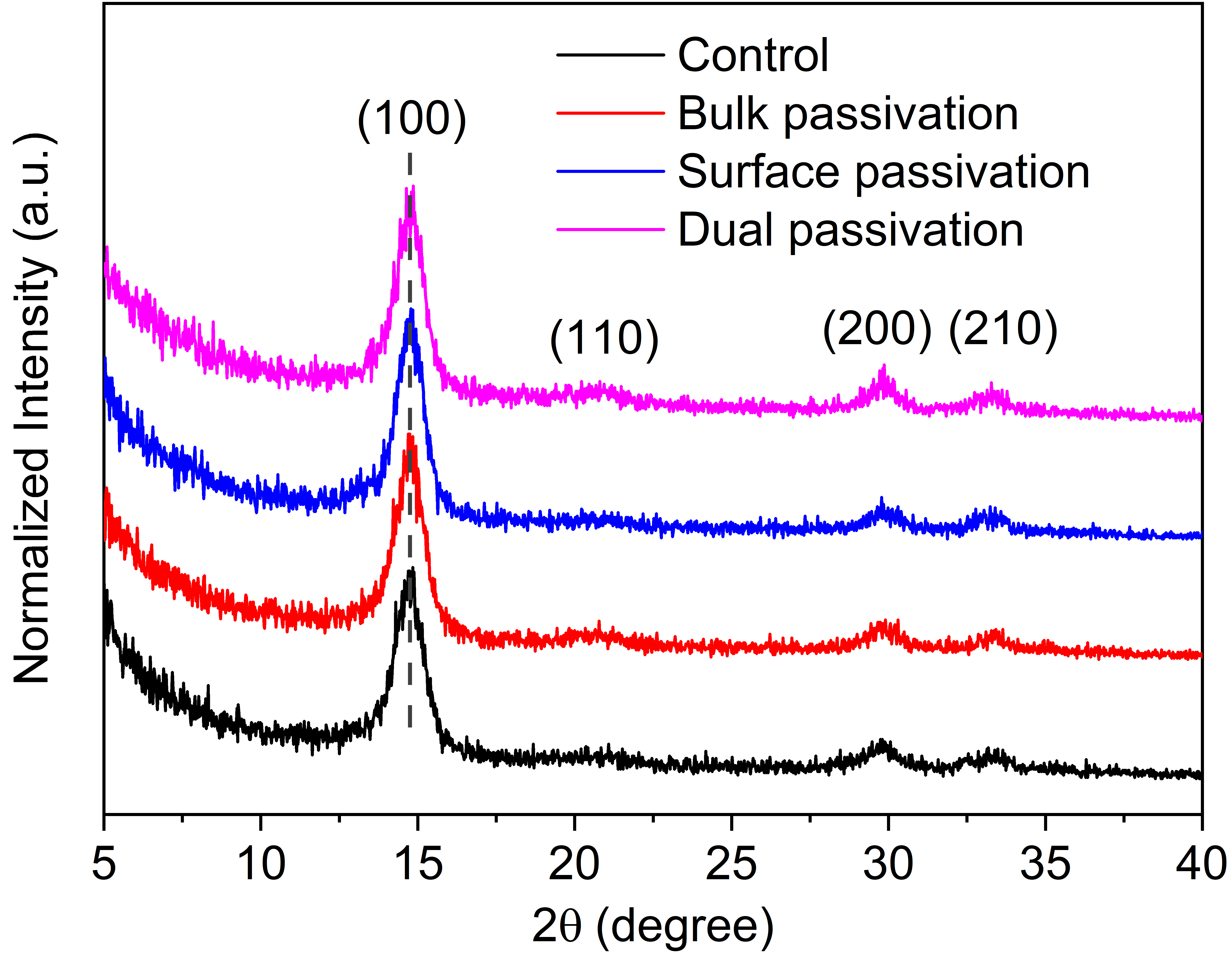


**Fig. S4** XRD patterns of quasi-2D perovskite films without and with different passivation strategies.


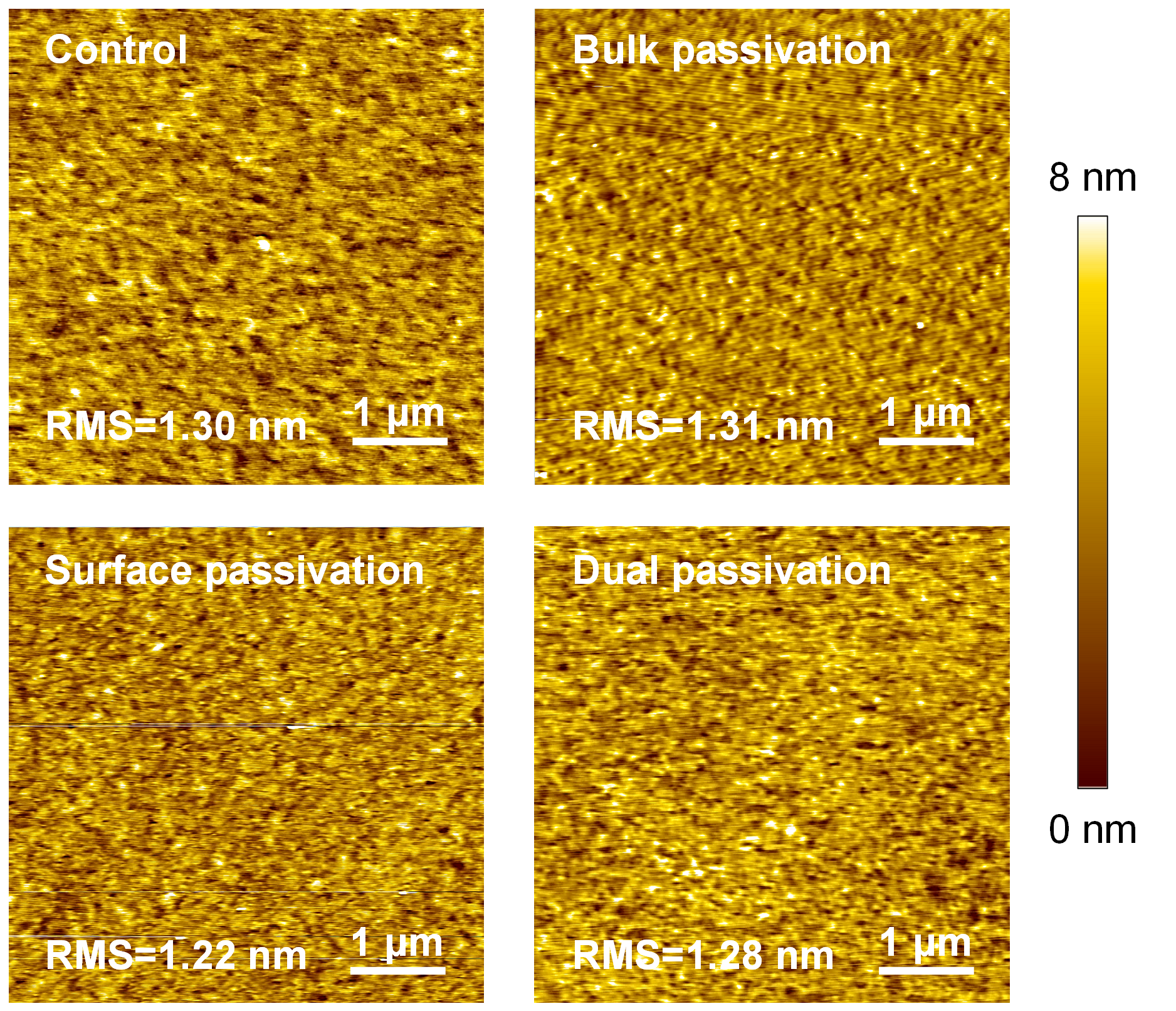


**Fig. S5** AFM images of quasi-2D perovskite films without and with different passivation strategies.


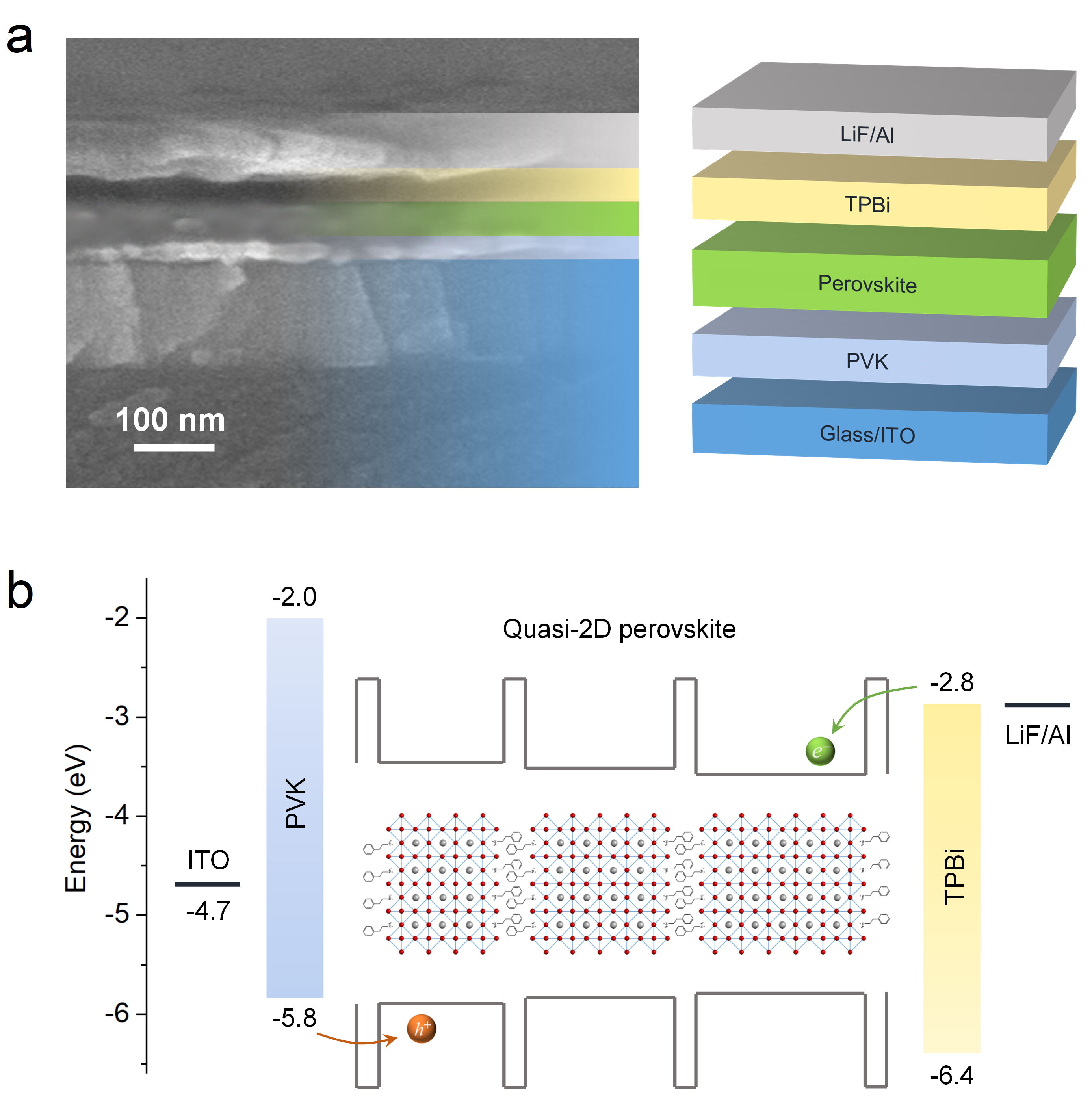


**Fig. S6** **a** Cross-sectional SEM image and **b** energy levels of fabricated PeLEDs.


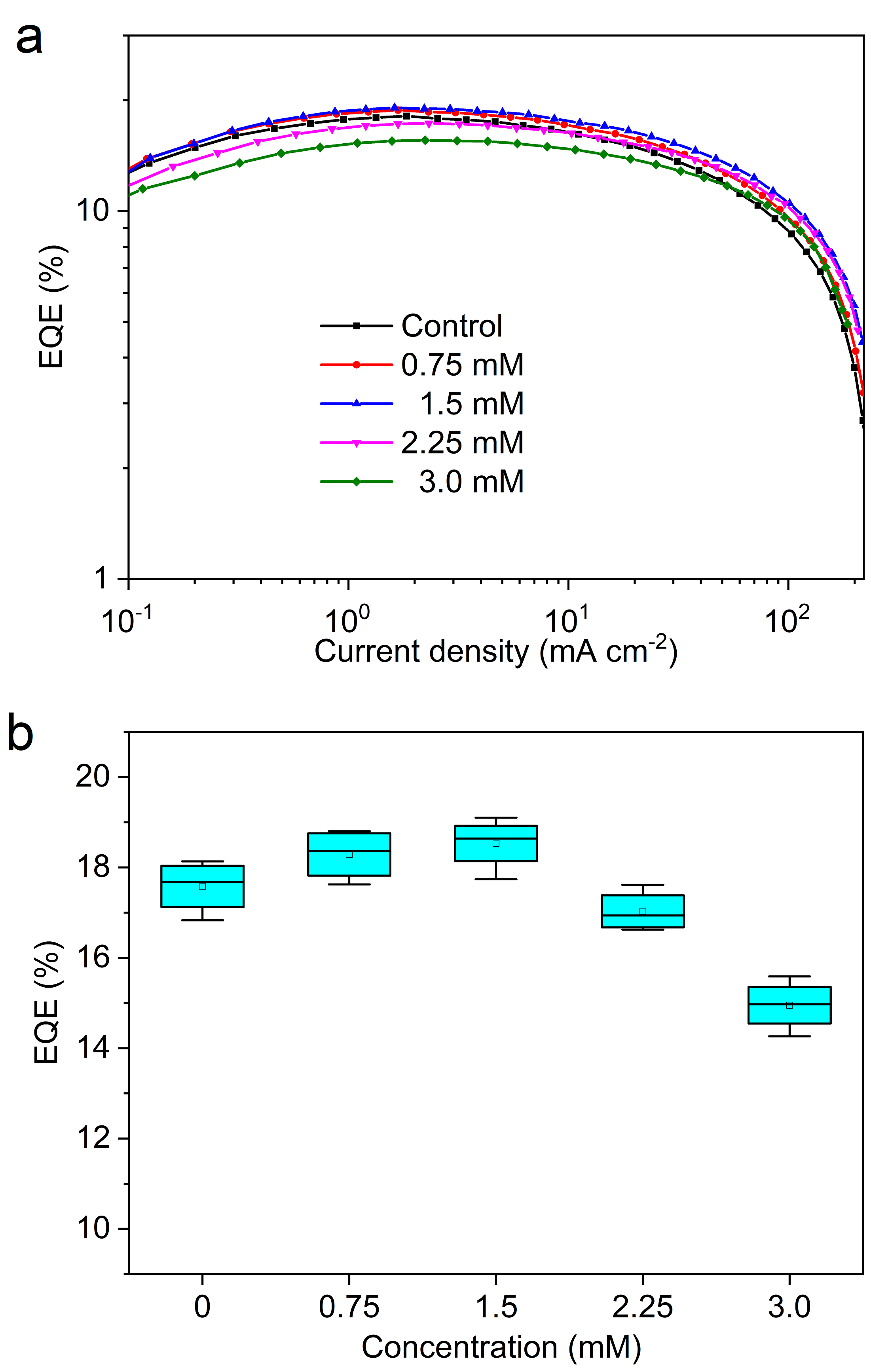


**Fig. S7** **a** Current density-EQE characteristics and **b** EQE distribution of PeLEDs without and with different concentrations of DBPF in perovskite precursor solution for bulk passivation.


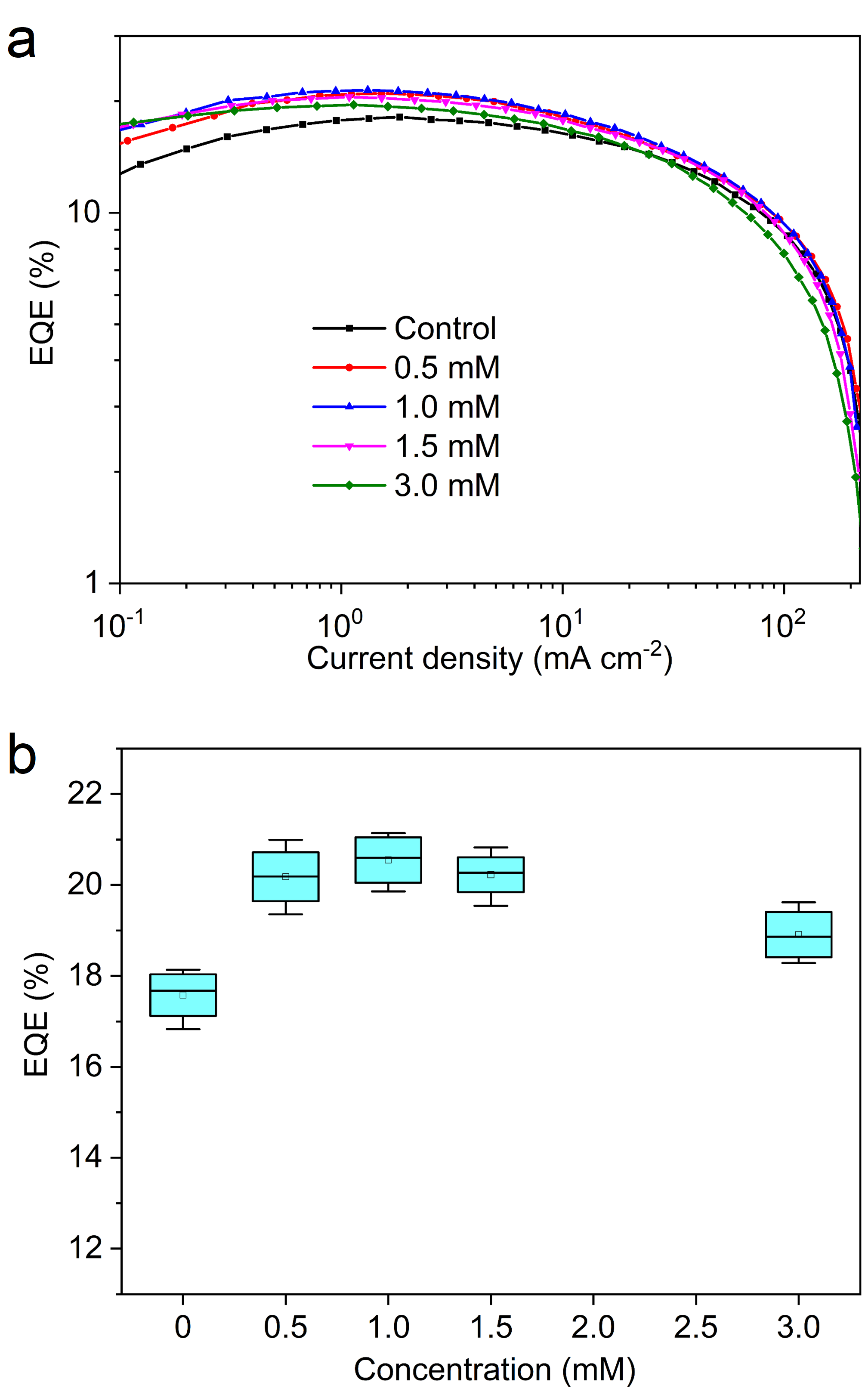


**Fig. S8** **a** Current density-EQE characteristics and **b** EQE distribution of PeLEDs without and with different concentrations of DBPF in antisolvent for surface passivation.


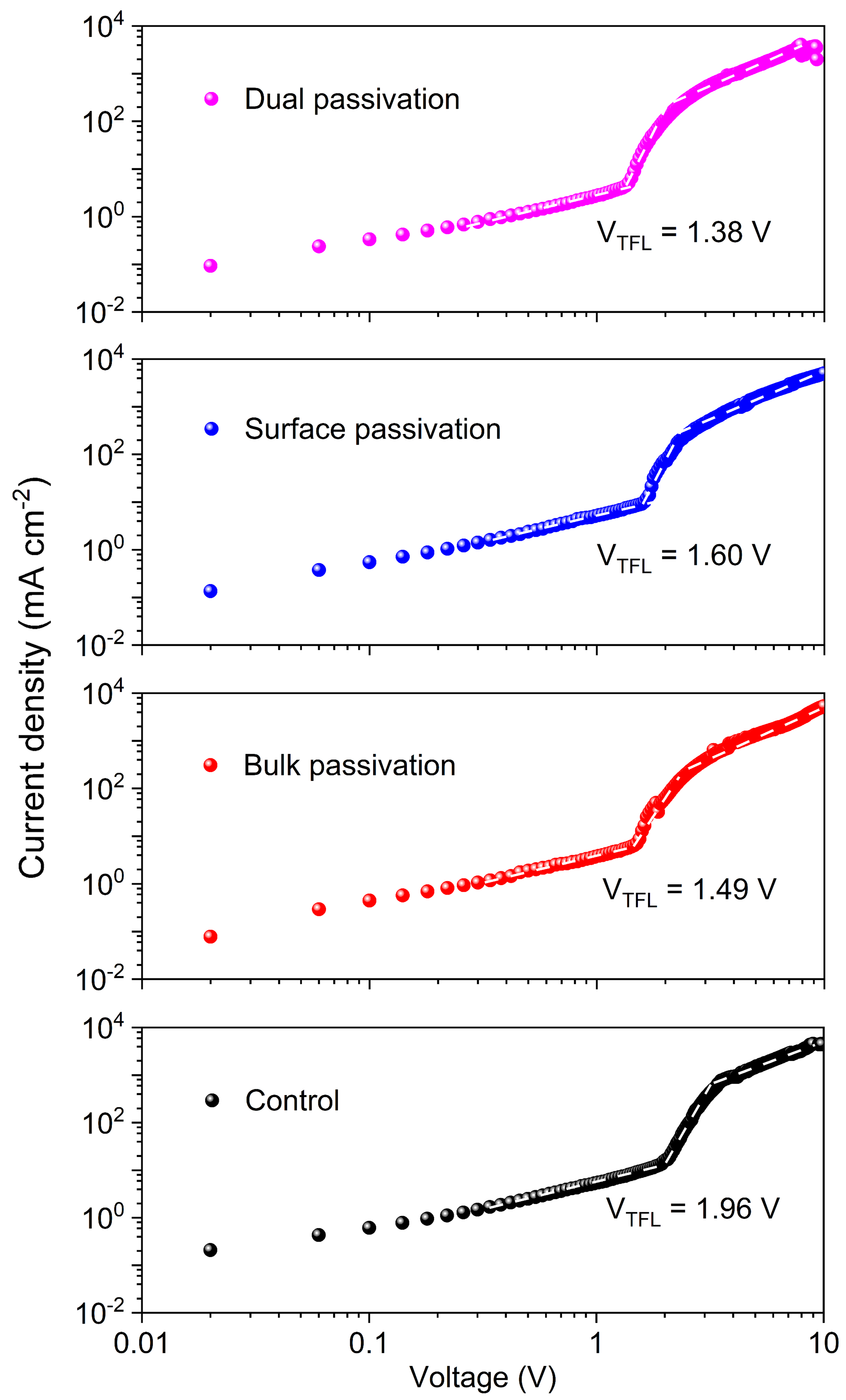


**Fig. S9** *J*-*V* curves of hole-only devices without and with different passivation strategies. The device structure is ITO/PVK/perovskite/MoO_3_/Ag. According to the formula of *V*_TFL_ = *eN*_t_*L*^2^/2*ε*_0_*ε*_r_, where *ε*_r_ is the dielectric constants of perovskite, *ε*_0_ is the vacuum permittivity, *L* is the thickness of perovskite film, *e* is the elementary charge, the trap density (*N*_t_) can be calculated by the trap-filled limit voltage (*V*_TFL_). Based on different passivation strategies, the *V*_TFL_ of devices is decreased, which indicates less traps in perovskite films.


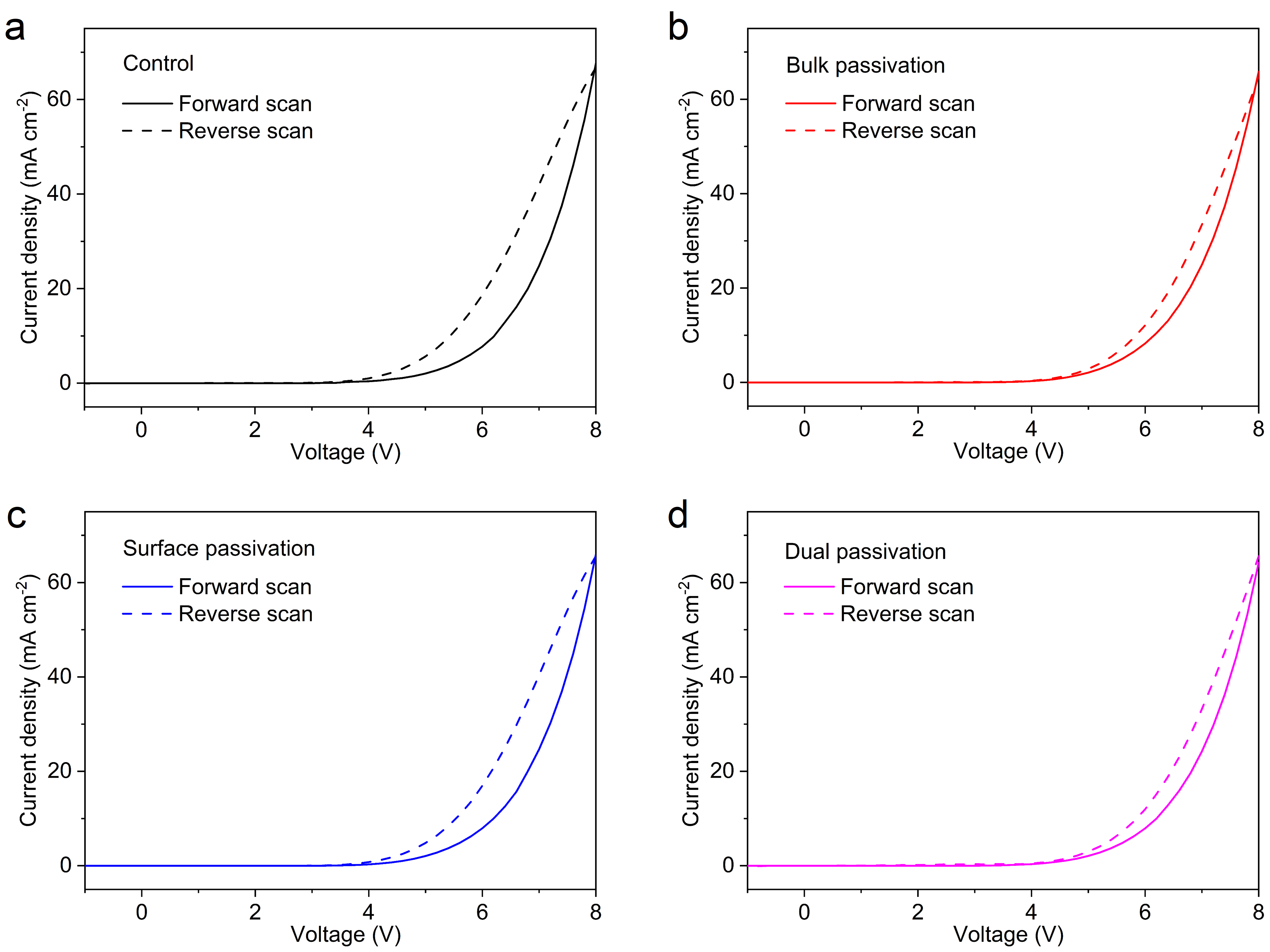


**Fig. S10** *J*-*V* curves of PeLEDs without and with different passivation strategies measured by forward and reverse scan.

**
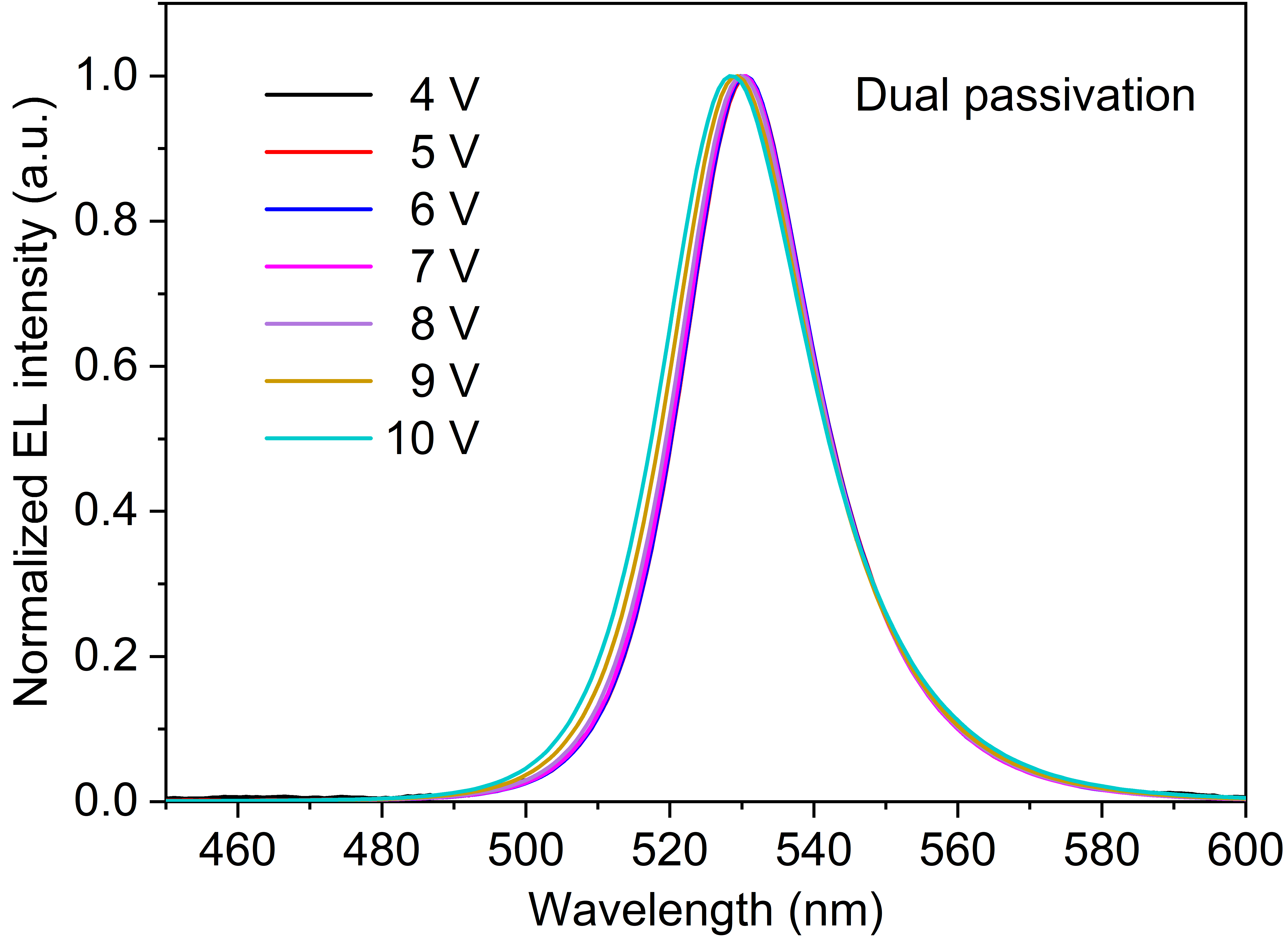
**

**Fig. S11** EL spectra of device with dual passivation under different applied bias.

**
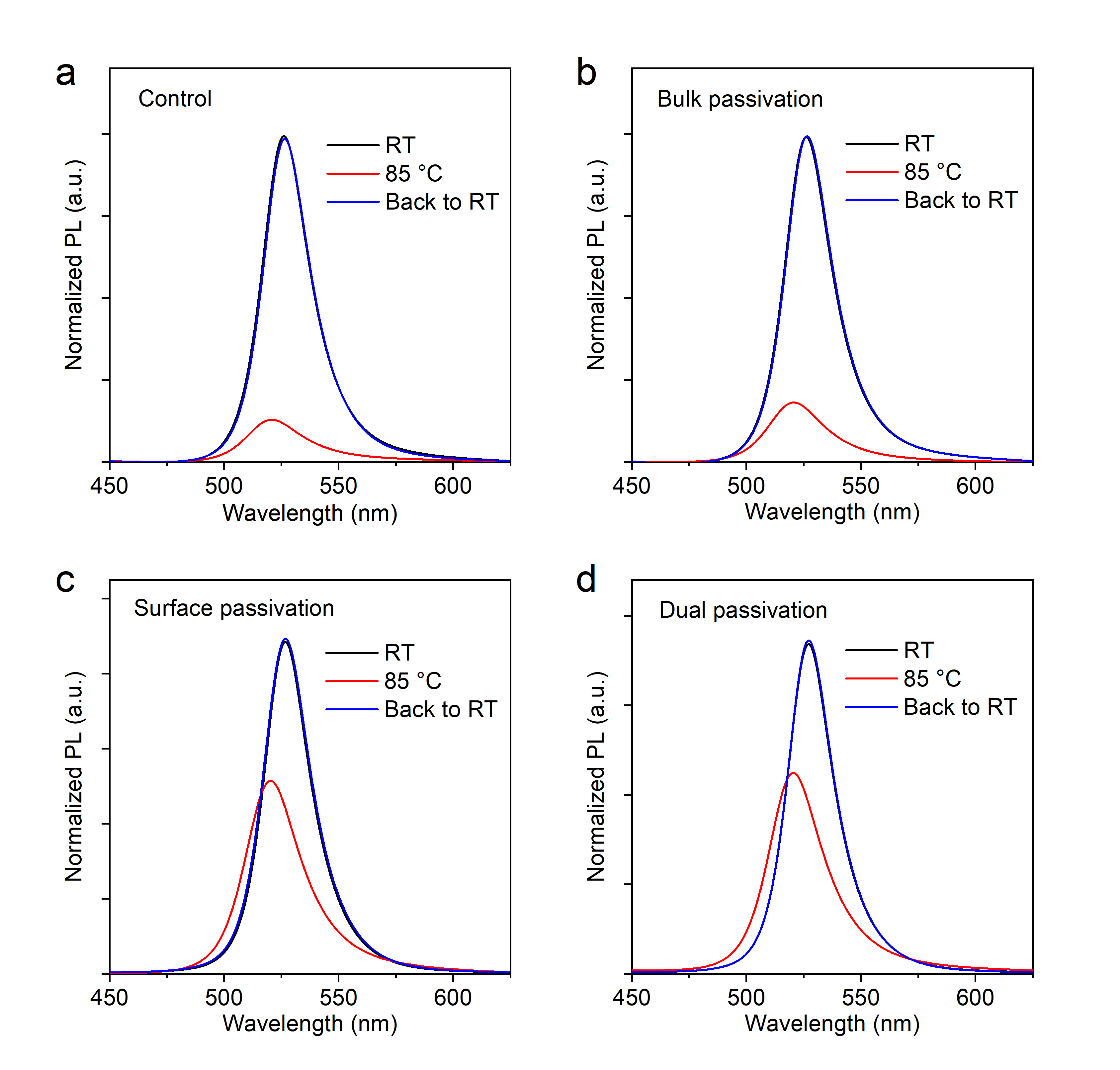
**

**Fig. S12** PL spectra of quasi-2D perovskite films without and with different passivation strategies at RT, annealing to 85 °C, and cooling down to RT, respectively.


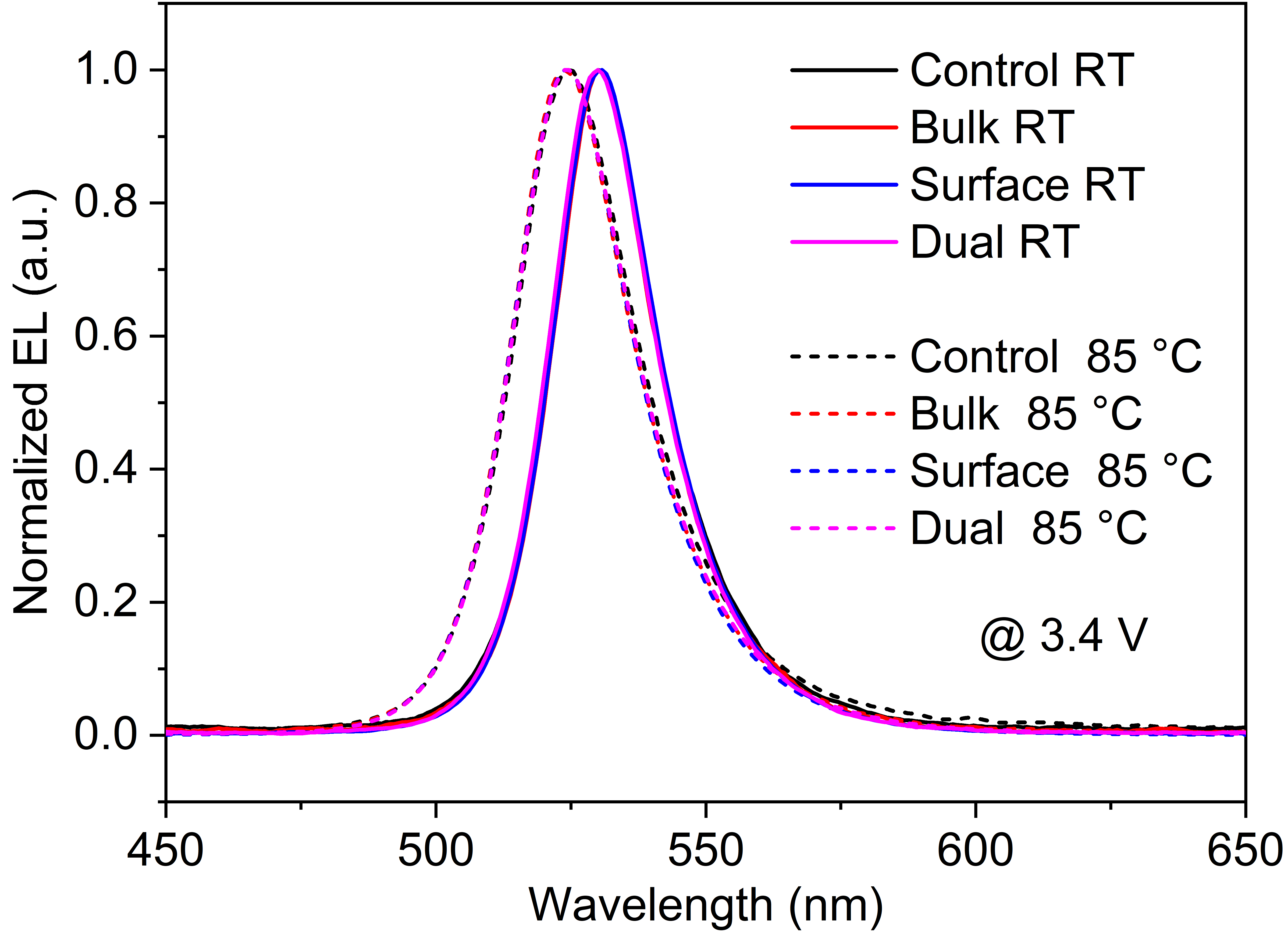


**Fig. S13** EL spectra of PeLEDs without and with different passivation strategies measured at RT and 85 °C.


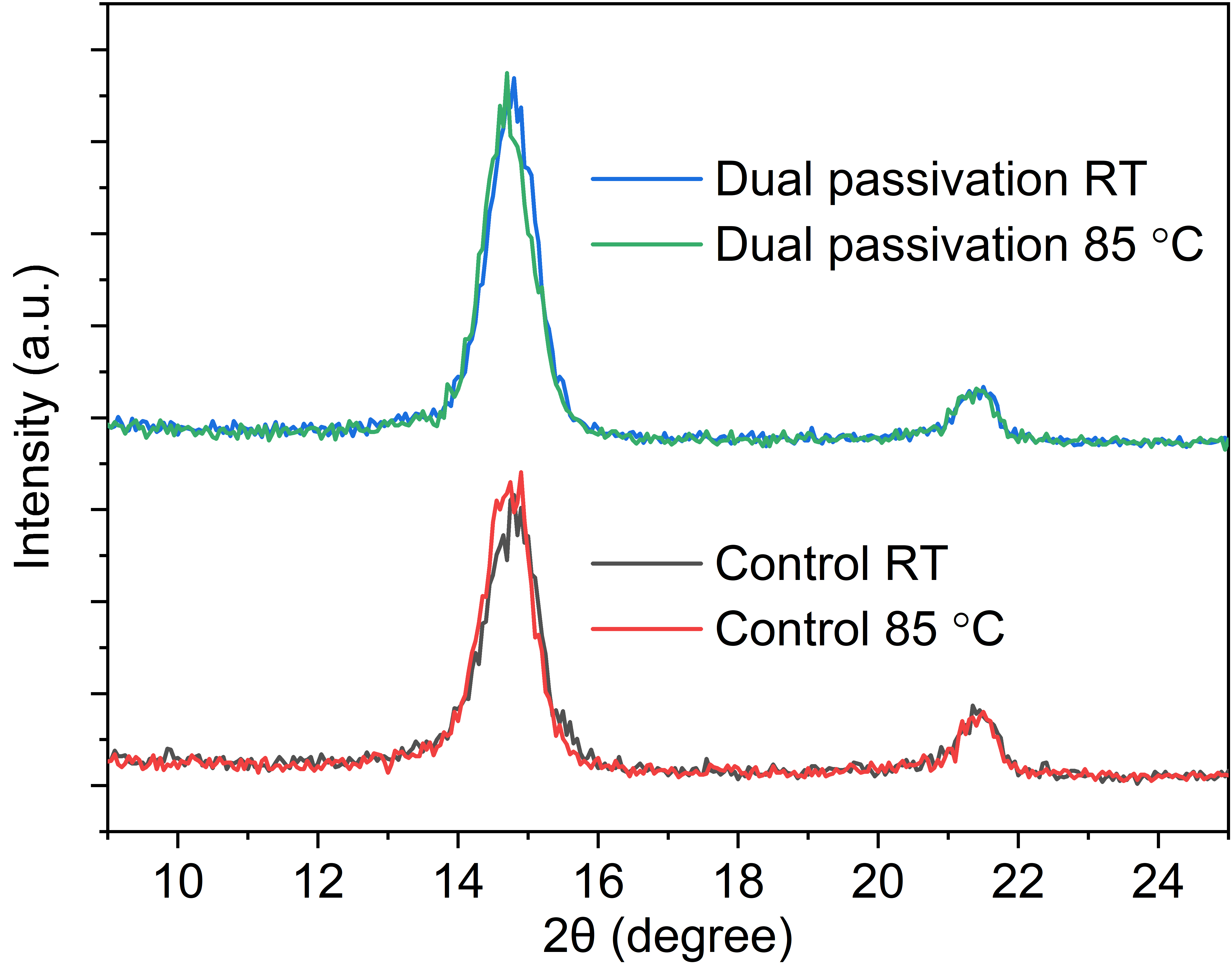


**Fig. S14** XRD measurements of control and dual passivation films at RT and 85 °C.


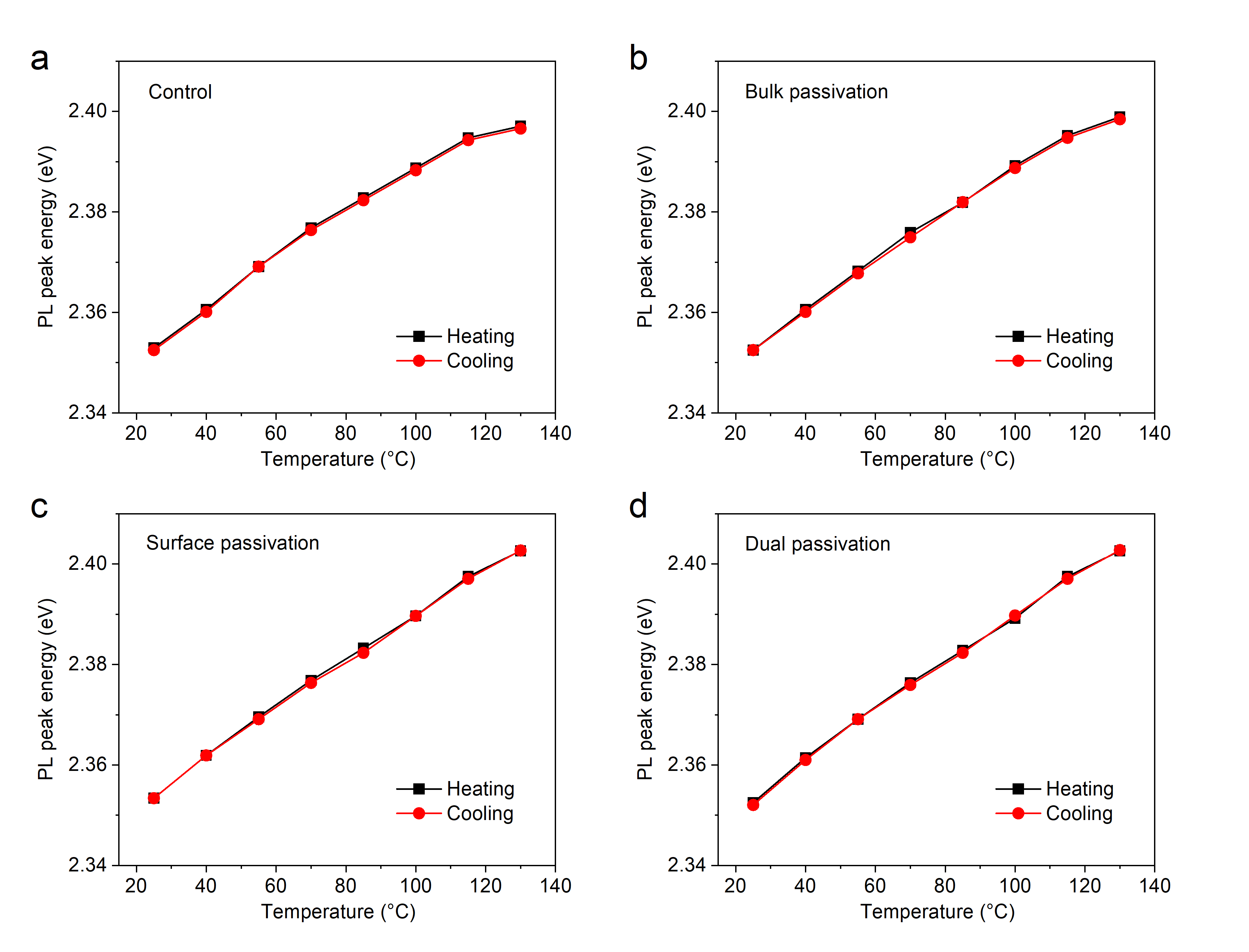


**Fig. S15** Plots of temperature-dependent PL peak energy of perovskite films without and with different passivation strategies for both heating and cooling cycle.

**
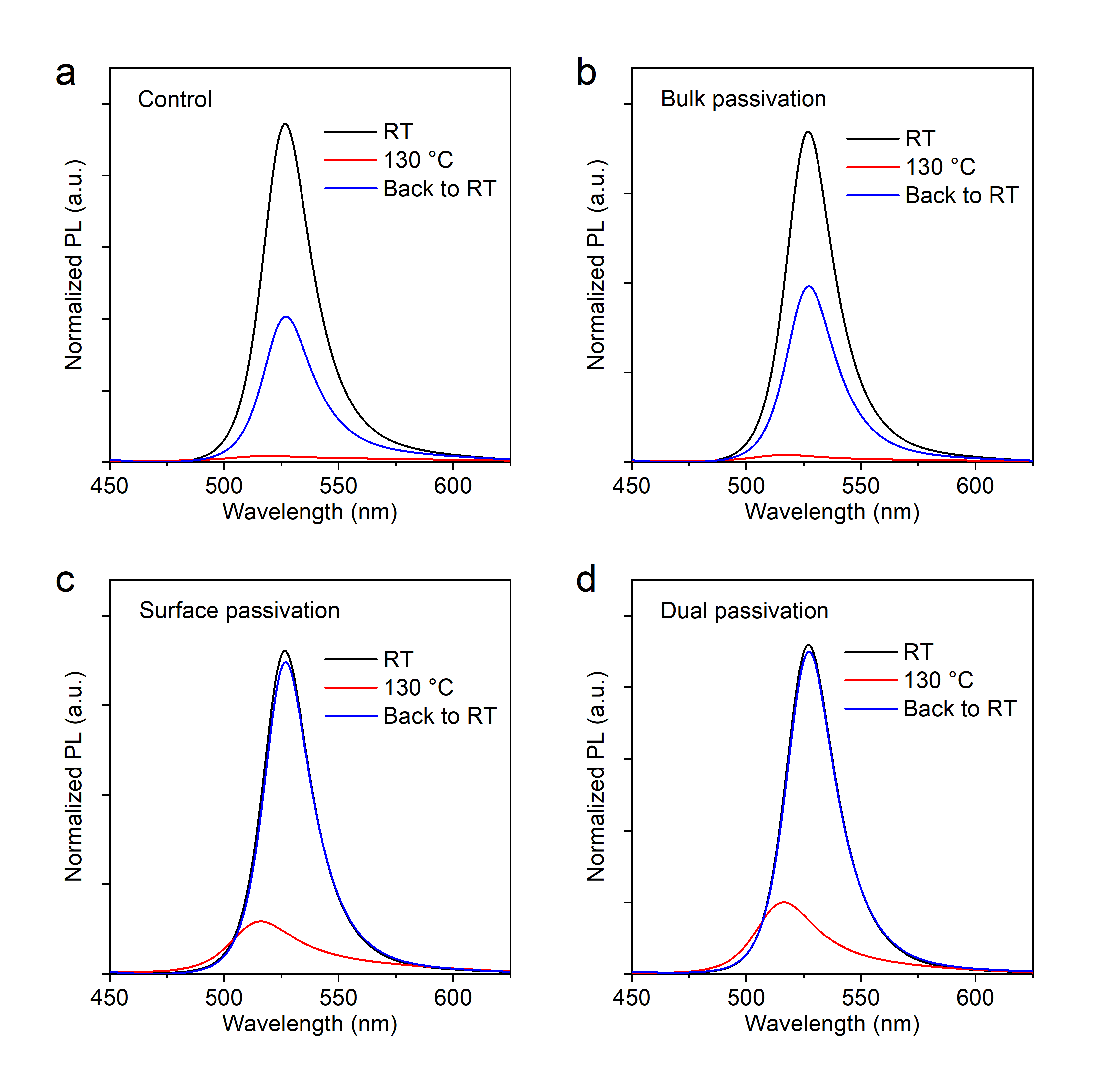
**

**Fig. S16** PL spectra of quasi-2D perovskite films without and with different passivation strategies at RT, annealing to 130 °C, and cooling down to RT, respectively.


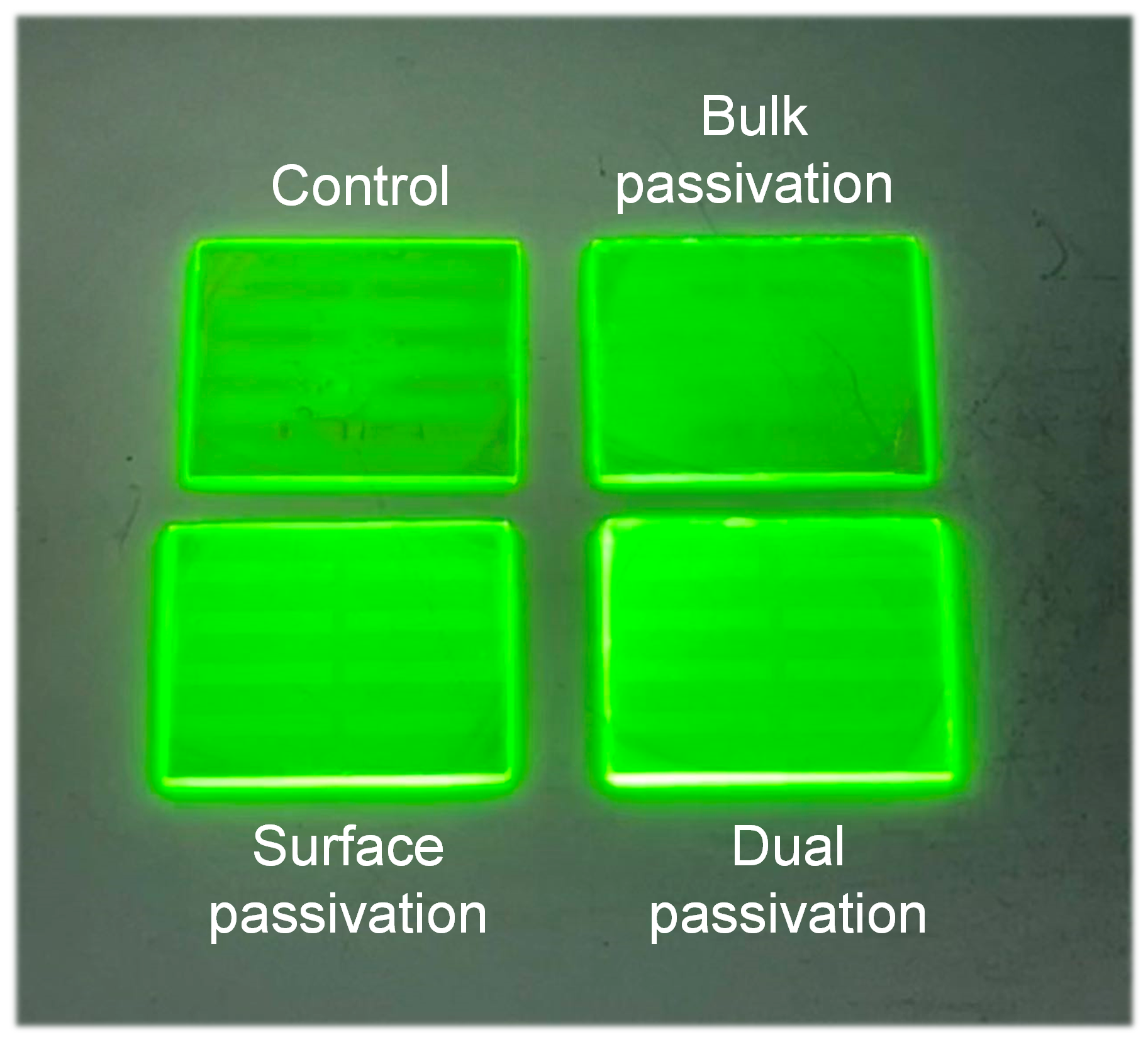


**Fig. S17** Photographs of quasi-2D films without and with different passivation strategies after returning to RT from 130 °C.


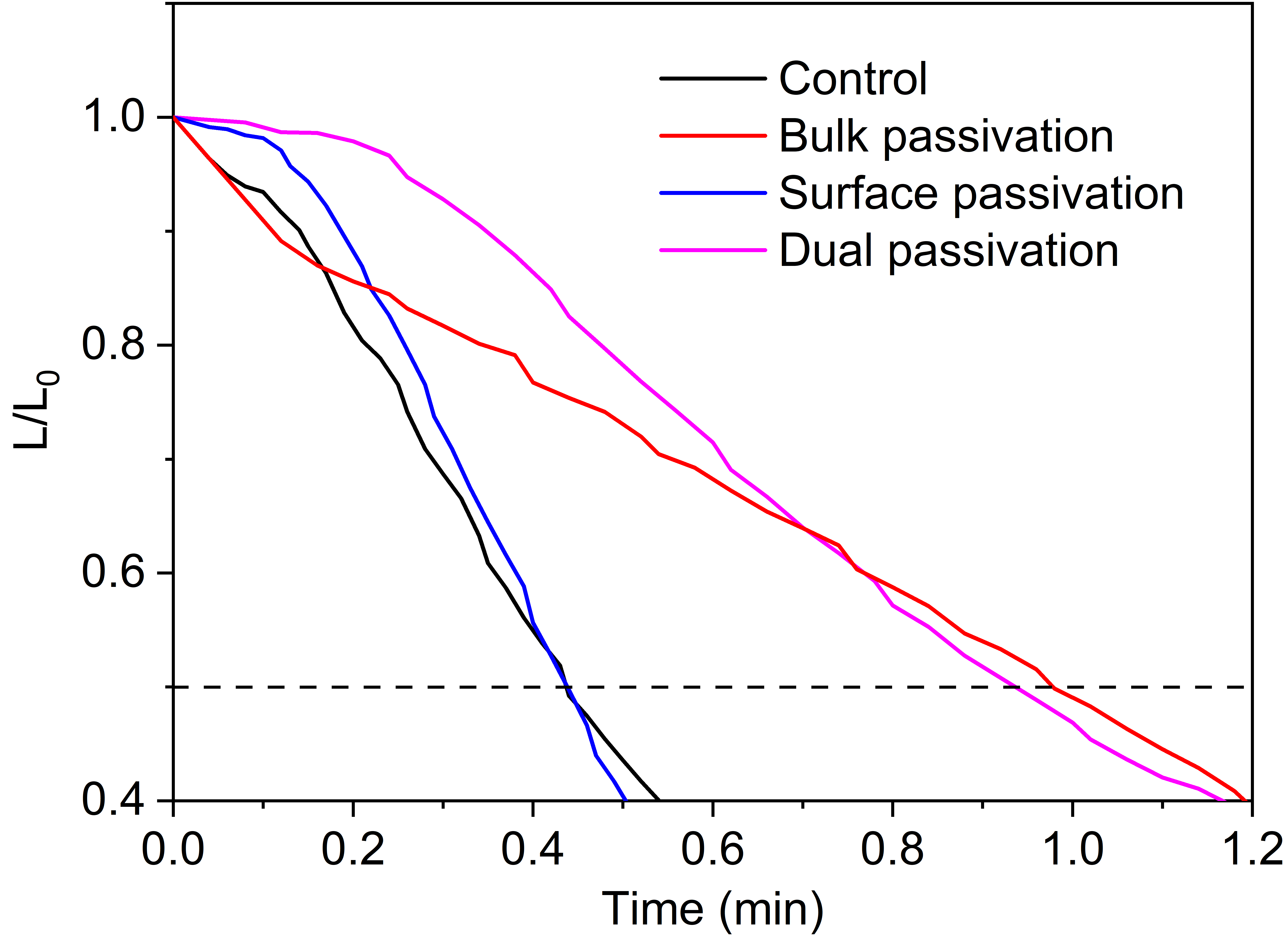


**Fig. S18** Operation stability of PeLEDs at 85 °C.


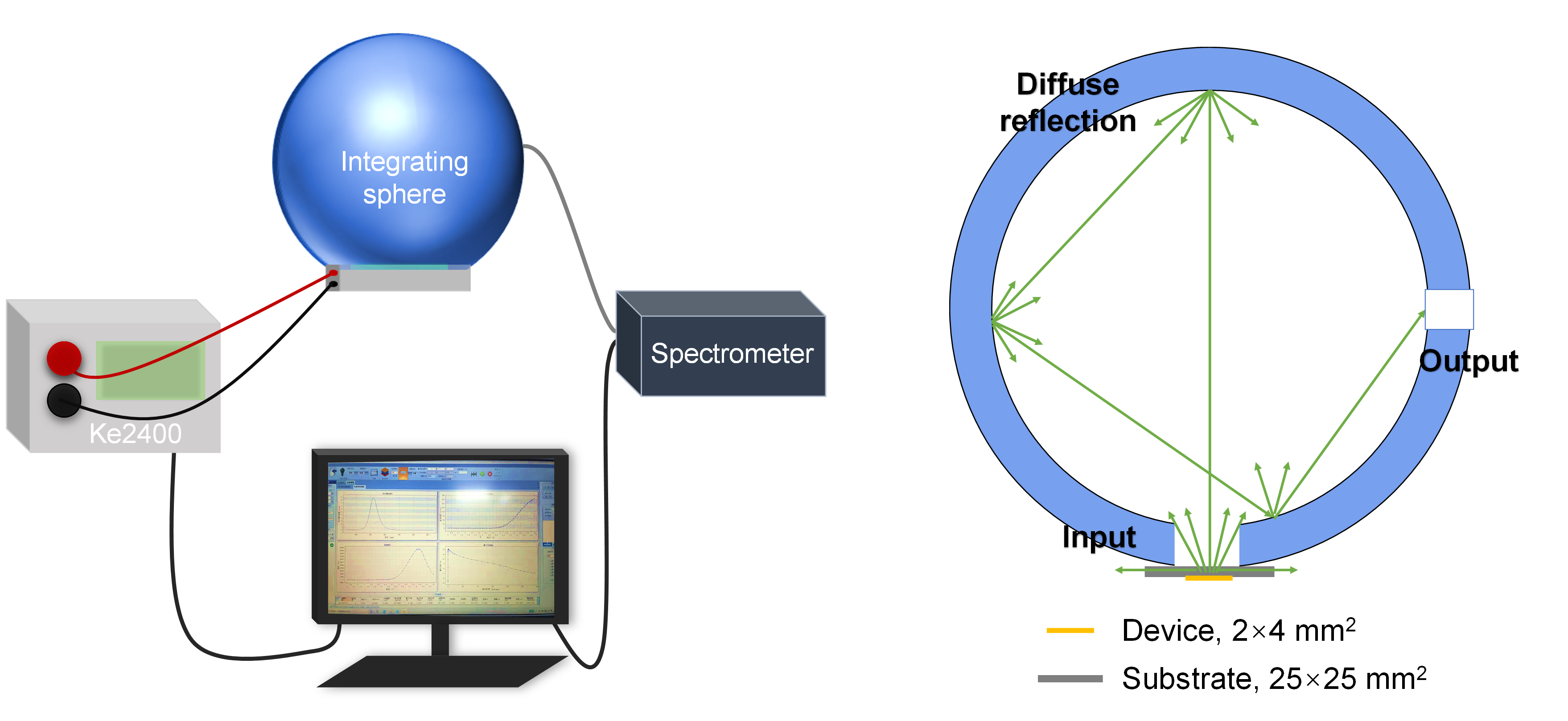


**Fig. S19** Schematic diagram of device characteristics test system.

**Table S1** Performance metrics of PeLEDs based on different passivation strategies at RT.

| Sample | Turn-on voltage (V) | EQE (%) (average/max.) | Max. Luminance (cd m^−2^) | Operation lifetime (min) |
| --- | --- | --- | --- | --- |
| Control | 3 | 17.0/18.1 | 4.9 × 10^4^ | 26 |
| Bulk passivation | 3 | 17.8/19.3 | 5.3 × 10^4^ | 54 |
| Surface passivation | 3 | 20.5/21.5 | 5.1 × 10^4^ | 28 |
| Dual passivation | 3 | 20.9/22.2 | 5.4 × 10^4^ | 53 |
